# Molecular phylogeny of the *Trechus brucki* group, with description of two new species from the Pyreneo-Cantabrian area (France, Spain) (Coleoptera, Carabidae, Trechinae)

**DOI:** 10.3897/zookeys.217.3136

**Published:** 2012-08-28

**Authors:** Arnaud Faille, Charles Bourdeau, Javier Fresneda

**Affiliations:** 1 Zoologische Staatssammlung München, Münchhausenstraße 21, 81247 Munich, Germany; 2 5 chemin Fournier-Haut, F–31320 Rebigue, France; 3 Ca de Massa, 25526 Llesp – El Pont de Suert, Lleida, Spain; 4 Museu de Ciències Naturals (Zoologia), Passeig Picasso s/n, 08003 Barcelona, Spain

**Keywords:** Carabidae, Trechini, *Trechus brucki* group, new species, molecular phylogeny, subterranean environment, Pyrenees, France, Spain

## Abstract

A molecular phylogeny of the species from the *Trechus brucki* clade (previously *Trechus uhagoni* group)based on fragments of four mitochondrial genes and one nuclear gene is given. We describe *Trechus (Trechus) bouilloni* **sp. n.** from the western pre–Pyrenees: Sierras de Urbasa–Andía, Navarra, Spain. The species was collected in mesovoid shallow substratum (mss), a subterranean environment. Molecular as well as morphological evidences demonstrate that the new species belongs to the *Trechus brucki* clade. A narrow endemic species of high altitude in western French Pyrenees merged with *Trechus brucki* Fairmaire, 1862a, *Trechus bruckoides* **sp. n.**, is described. A lectotype is designated for *Trechus brucki* and *Trechus planiusculus* Fairmaire, 1862b (junior synonym of *Trechus brucki*). The species group is redefined based on molecular and morphological characters, and renamed as the *brucki* group, as *Trechus brucki* was the first described species of the clade. A unique synapomorphy of the male genitalia, a characteristic secondary sclerotization of the sperm duct, which is shared by all the species of the *brucki* group sensu novo, is described and illustrated. The *Trechus brucki* group sensu novo is composed of *Trechus beusti* (Schaufuss, 1863), *Trechus bouilloni* **sp. n.**, *Trechus brucki*, *Trechus bruckoides* **sp. n.**, *Trechus grenieri* Pandellé, 1867,* T. uhagoni uhagoni* Crotch, 1869,* T. uhagoni ruteri* Colas, 1935 and *Trechus pieltaini* Jeannel, 1920. We discuss the taxonomy of the group and provide illustrations of structures showing the differences between the species, along with distribution data and biogeographical comments.

## Introduction

The genus *Trechus* (Coleoptera, Carabidae, Trechinae) includes more than 800 species, most of them in the Palearctic area (Moravec et al.2003; Lorenz 2005). This genus is known to contain many wingless short range endemic species (Jeannel 1927, Casale and Laneyrie 1982, Schmidt 2009) and is currently understood as polyphyletic (Faille et al. 2010a, 2011a).

Jeannel (1927) gathered seven species from the *uhagoni* group distributed from the French slope of the Pyrenees to the Cantabrian area: *Trechus bonvouloiri* Pandellé, 1867 (France: Hautes–Pyrénées), *Trechus bordei* Peyerimhoff, 1909 (France: Pyrénées–Atlantiques), *Trechus brucki* Fairmaire, 1862 (France: Hautes–Pyrénées, Pyrénées–Atlantiques), *Trechus grenieri* Pandellé, 1867 (France: Hautes–Pyrénées), *Trechus navaricus* Vuillefroy, 1867 (France: Pyrénées–Atlantiques), *Trechus sharpi* Jeannel, 1921 (Spain: Cantabria) and *Trechus uhagoni* Crotch, 1869 (Spain: Navarra). This group of species was also considered close to the group of *Trechus angusticollis* Kiesenwetter, 1850, a Pyreneo–Cantabrian group with nine species, all apterous, orophilous or troglobitic.

In this paper we describe a species collected by traps in a MSS (mesovoid shallow substratum, “Milieu Souterrain Superficiel” sensu Juberthie et al. 1980, Giachino and Vailati 2010) in the Sierras de Urbasa–Andía (Western pre–Pyrenees, Navarra, Spain) and a second orophilic species from the French Central Pyrenees. We study the phylogenetic relationships of the new species and provide a molecular phylogeny of the group, including all known species but four.

### Historical background

*Trechus uhagonii* was described by Crotch in 1869 and dedicated to S. de Uhagon with whom he visited caves in the Alsasua area in June 1869. *Trechus bruckii* was first described under the name *Trechus politus* by Fairmaire (1862b). He renamed it one year later to *bruckii* because *politus* was already in use for an American species. The two names were corrected to *uhagoni* and *brucki* by subsequent authors, and recently renamed *uhagonii* and *bruckii* in catalogues (Lorenz 1998, 2005, Moravec et al. 2003, Queinnec and Ollivier 2011). As the names *uhagonii* and *bruckii* have not been used since their description, we choose to keep the prevailing usage of *uhagoni* and *brucki* in accordance with the article 33.3.1 of the International Code of Zoological Nomenclature on incorrect subsequent spellings (ICZN 1999).

In the Monographie des Trechinae, Jeannel (1927) erected the *uhagoni* group for the seven species of *Trechus* from the Pyreneo–Cantabrian area quoted above. Español (1970) described *Trechus ortizi* from a cave of Burgos province (Spain), and included it in the *uhagoni* group, close to *Trechus bordei*.

The *uhagoni* group sensu Jeannel (1927), although poorly defined morphologically, was enriched with 5 species by Casale and Laneyrie (1982) in their catalogue of species of world Trechinae: *Trechus pecoudi* Colas & Gaudin, 1935 (described first as a subspecies of *Trechus brucki*), *Trechus ortizi*, *Trechus escalerae* Abeille de Perrin, 1903 (considered by Jeannel (1927) to belong to the *Trechus angusticollis* group), *Trechus enigmaticus* Coiffait, 1971 and *Trechus aubryi* Coiffait, 1953. *Trechus uhagoni* was here considered as subspecies of *Trechus grenieri*. By describing *Trechus baztanensis* from a cave of Navarra, Dupré (1991) suggested that the peculiar genital morphology of *Trechus bordei*, *Trechus navaricus*, *Trechus bonvouloiri* and the new species should lead to their removal from the *uhagoni* group, and he created the *bonvouloiri* group for these species, opinion followed by Queinnec and Ollivier (2011). Toribio and Rodríguez (1997) added one species from Cantabria to the *uhagoni* group, *Trechus carrilloi*, a species collected in the MSS. Sciaky (1998) described *Trechus jeannei* from Cantabria, and included it in the *uhagoni* group, close to *Trechus bordei* and *Trechus ortizi*. Hernando (2002) described *Trechus comasi* from a cave of Navarra and suggested that it should be considered as sister species of *Trechus brucki*. Molecular and morphological evidence suggest that this species should be removed from the *uhagoni* group (Faille et al. 2010a, 2011a, Ortuño and Arribas 2010). Ortuño and Toribio (2005) described a new species of *Trechus* belonging to another group of species, indicating that 11 species belong to the *uhagoni* group in the Iberian Peninsula. Reboleira et al. (2010) considered that 10 species belong to this group in the Peninsula, without providing the list of taxa included.

*Trechus brucki* is an alpine species located at high altitude in the central and western Pyrenees, and it is until now not recorded from the Spanish slope of the chain (Serrano 2003). Colas and Gaudin described *Trechus pecoudi* in 1935 from the western Pyrenees (Pic d´Orhy) as a subspecies of *Trechus brucki*. Coiffait (1952) described 3 subspecies of *Trechus brucki*; *Trechus brucki vandeli*, *Trechus brucki truilheti* and *Trechus brucki microthorax*. The subspecies *vandeli* and *truilheti* were later related to *Trechus pecoudi* (Casale & Laneyrie, 1982) so that *Trechus pecoudi* counts three subspecies in the Catalogue of Palearctic Coleoptera (Moravec et al. 2003). Queinnec and Ollivier (2011) considered *Trechus pecoudi* as a subspecies of *brucki* restricted in the Anie and Orhy massifs, whereas the subspecies *vandeli*, described from Anie, was considered a synonym of *Trechus brucki brucki* together with the subspecies *truilheti* and *microthorax*.

## Materials and methods

### Taxon sampling, Morphological study, DNA extraction and sequencing

Specimens were collected by hand or by means of pitfall traps containing water saturated in salt or propylene glycol, known to preserve DNA (Rubink et al. 2003, López and Oromí 2010) (Table 1). The protocol is detailed in Faille et al. (2010b):

Extractions of single specimens were non–destructive, using the DNeasy Tissue Kit (Qiagen GmbH, Hilden, Germany). After extraction, specimens were mounted on cards and genitalia stored in water–soluble dimethyl hydantoin formaldehyde resin (DMHF) on transparent cards, pinned beneath the specimen. Vouchers and DNA samples are kept in the collections of ZSM, IBE and MNHN.

We included examples of most species of the *Trechus uhagoni* group, with the exception of *Trechus bruckoides* sp. n., *Trechus carrilloi* and *Trechus sharpi* and some examples of *Trechus* of the *angusticollis* group sensu Jeannel (1927) and Casale and Laneyrie (1982) (Table 1). The tree was rooted with *Aphaenops leschenaulti* Bonvouloir, 1862, which is known to belong to a different group of Pyrenean Trechini (Jeannel 1927, Faille et al. 2010a).

We amplified fragments of four mitochondrial genes: 3’ end of cytochrome c oxidase subunit (*cox1*); a single fragment including the 3’ end of the large ribosomal unit (*rrnL*), the whole tRNA–Leu gene (*trnL*) and the 5’ end of the NADH dehydrogenase 1 (*nad1*);and one nuclear gene (internal fragment of the large ribosomal unit 28S rRNA, *LSU*) (see Table 2 for primers used). Sequences were assembled and edited using Sequencher TM 4.8 (Gene Codes, Inc., Ann Arbor, MI). Parts of the sequences for 14 of the species were taken from Faille et al. (2010a) and Faille et al. 2011a (Table 1).

New sequences have been deposited in the EMBL database with Accession Numbers HE817887–HE817940 (Table 1).

**Table 1.** Sequenced specimens, with localities, collectors, codes and sequence accession numbers (unpublished sequences in bold).

**sp**	**locality**	**collector**	**code**	**LSU**	**cox1**	**rrnL**	**trnL**	**NAD1**
***Aphaenops* Bonvouloir, 1862**								
*Aphaenops leschenaulti* Bonvouloir, 1861	Grotte de Castelmouly – Bagnères–de–Bigorre (France–65)	C. Bourdeau, P. Déliot, A. Faille	MNHN–AF1	GQ293593	**HE817919**	GQ293739	GQ293757	GQ293822
***Trechus* Clairville, 1806**								
*Trechus grenieri* Pandellé, 1867	Résurgence de la Hèche, Fréchet–Aure (France–65)	J.P. Besson, C. Bourdeau, A. Faille	ZSM–L13	**HE817904**	**HE817920**	**HE817887**	**HE817887**	**HE817887**
*Trechus brucki* Fairmaire, 1862	Pic du Gabizos, Arrens (France–65)	C. Bourdeau	ZSM–L329	**HE817906**	**HE817921**	**HE817888**	**HE817888**	**HE817888**
*Trechus brucki* Fairmaire, 1862	Pic du Gabizos, Arrens (France–65)	C. Bourdeau	ZSM–L329b	**HE817907**		**HE817889**	**HE817889**	**HE817889**
*Trechus brucki* Fairmaire, 1862	Pic de Sesques, Laruns (France–64)	C. Bourdeau	ZSM–L446	**HE817905**	**HE817922**	**HE817890**	**HE817890**	**HE817890**
*Trechus brucki* Fairmaire, 1862	Pic de Gaziès, Laruns (France–64)	C. Bourdeau	ZSM–L190	**HE817908**	**HE817923**	**HE817891**	**HE817891**	**HE817891**
*Trechus brucki* Fairmaire, 1862	Caperan d´Anéou, Laruns (France–64)	C. Bourdeau	ZSM–L449	**HE817909**	**HE817924**	**HE817892**	**HE817892**	**HE817892**
*Trechus uhagoni* Crotch, 1869	Cueva de Orobe – Alsasúa (Spain–Navarra)	C. Bourdeau, J. Fresneda	ZSM–L161	**HE817910**	**HE817925**	**HE817893**	**HE817893**	**HE817893**
*Trechus bouilloni* Faille, Bourdeau & Fresneda, sp. n.	Puerto de Lizarraga, Lizarraga (Spain–Navarra)	C. Bourdeau, J. Fresneda	ZSM_L201b	**HE817911**	**HE817926**	**HE817894**	**HE817894**	**HE817894**
*Trechus bouilloni* Faille, Bourdeau & Fresneda, sp. n.	Puerto de Lizarraga, Lizarraga (Spain–Navarra)	C. Bourdeau, J. Fresneda	ZSM_L201t		**HE817927**	**HE817895**	**HE817895**	**HE817895**
*Trechus beusti* (Schaufuss 1863)	Cueva de San Adrián, Zegama (Spain–Guipúzcoa)	C. Bourdeau, J. Fresneda	ZSM–L199	**HE817912**	**HE817928**	**HE817896**	**HE817896**	**HE817896**
*Trechus pieltaini* Jeannel, 1920	Cueva de Mairuelegorreta, Gorbea (Spain–Álava)	C. Bourdeau	ZSM–L395	**HE817913**	**HE817929**	**HE817897**	**HE817897**	**HE817897**
*Trechus navaricus* (Vuillefroy, 1869)	Grotte de Sare – Sare (France–64)	C. Bourdeau	MNHN–AF103	GQ293603	GQ293687	**FR729578**	**FR729578**	**FR729578**
*Trechus bordei* Peyerimhoff, 1909	Grotte d´Ayssaguer – Larrau (France–64)	C. Bourdeau, P. Déliot, A. Faille	MNHN–TBA	**HE817914**	**HE817930**	**HE817898**	**HE817898**	**HE817898**
*Trechus bonvouloiri* Pandellé, 1867	Pic de Montaigu – Baudéan (France – 65)	C. Bourdeau	ZSM–L218	**HE817915**	**HE817931**	**HE817899**	**HE817899**	**HE817899**
*Trechus abeillei* Pandellé, 1872	Cirque d´Anglade Couflens (France–09)	C. Vanderbergh	ZSM–L15	**HE817916**	**HE817932**	**HE817900**	**HE817900**	**HE817900**
*Trechus distinctus* Fairmaire & Laboulbène, 1854	Col Sobe Ariel – Laruns (France–64)	C. Bourdeau	ZSM–L216	**HE817917**	**HE817933**	**HE817901**	**HE817901**	**HE817901**
*Trechus aubryi* Coiffait, 1953	Cirque d´Anglade Couflens (France–09)	B. Junger	ZSM–L370		**HE817934**	**HE817902**	**HE817902**	**HE817902**
*Trechus jeannei* Sciaky, 1998	Bosque de Saja, Saja (Spain–Cantabria)	C. Bourdeau	ZSM–L516	**HE817918**		**HE817903**	**HE817903**	**HE817903**
*Trechus saxicola* Putzeys, 1870	Braña Caballo – Piedrafita (Spain–León)	C. Bourdeau, P. Déliot, A. Faille	MNHN–AF100	GQ293614	**HE817935**	FR729577	FR729577	FR729577
*Trechus escalerae* Abeille de Perrin, 1903	Cueva de Porro Covañona – Covadonga (Spain–Asturias)	J.M. Salgado	MNHN–AF104	GQ293612	FR733912	GQ293731	GQ293793	GQ293839
*Trechus ceballosi* Mateu, 1953	Aven de Licie Etsaut – Lanne–en–Barétous (France–64)	C. Bourdeau, A. Faille	MNHN–AF128	GQ293610	FR733914	GQ293728	GQ293791	GQ293850
*Trechus distigma* Kiesenwetter, 1851	Aven de Nabails – Arthez d’Asson (France–64)	C. Bourdeau, P. Déliot, A. Faille	MNHN–AF94	GQ293611	**HE817936**	FR729575	FR729575	FR729575
*Trechus barnevillei* Pandellé, 1867	Cueva del Pis – Penilla, Santiurde de Toranzo (Spain–Cantabria)	C. Bourdeau, P. Déliot, A. Faille	MNHN–AF97	GQ293607	GQ293680	GQ293727	GQ293783	GQ293848
*Trechus obtusus* Erichson, 1837	Estrada de Nicho (Portugal–Madeira)	A. Arraiol	IBE–AF2	FR733997	**HE817937**	FR729579	FR729579	FR729579
*Trechus quadristriatus* (Schrank, 1781)	Collau de la Plana del Turbón – Egea (Spain– Huesca)	P. Déliot, A. Faille, J. Fresneda	MNHN–AF96	GQ293619	FR733908	GQ293743	GQ293745	GQ293841
*Trechus fulvus* Dejean, 1831	Cueva del Pis – Penilla, Santiurde de Toranzo (Spain–Cantabria)	C. Bourdeau, P. Déliot, A. Faille	MNHN–AF98	GQ293613	**HE817938**	GQ293729		
*Trechus martinezi* Jeannel, 1927	Cova de les Meravelles – Cocentaina (Spain– Alicante)	C. Andújar, P. Arribas, A. Faille	IBE–AF1	FR733996	**HE817939**	FR729576	FR729576	FR729576
*Trechus schaufussi ssp. comasi* Hernando, 2002	Cueva Basaula – Barindano (Spain–Navarra)	J. Fresneda	MNHN–AF127	GQ293617	**HE817940**	FR729580	FR729580	FR729580
***Apoduvalius* Jeannel, 1953**								
*Apoduvalius alberichae* Español, 1971	Cova de Agudir – Cardano de abajo – Palencia (Spain–Asturias)	J.M. Salgado	MNHN–AF105	GQ293618	GQ293632	GQ293732	GQ293794	GQ293840
*Apoduvalius anseriformis* Salgado et Peláez, 2004	Cueva de Entrecuevas – Caravia Alta (Spain– Palencia)	A. Cieslak, A. Faille, J. Fresneda, I. Ribera, J.M. Salgado	MNCN–AF2	FR733999	FR733916	FR729582	FR729582	FR729582

**Table 2.** Primers used in the study. F, forward; R, reverse.

**Gene**	**Name**	**Sense**	**Sequence**	**Reference**
*cox1*	Jerry (M202)	F	CAACATTTATTTTGATTTTTTGG	Simon et al. 1994
	Pat (M70)	R	TCCA(A)TGCACTAATCTGCCATATTA	Simon et al. 1994
	Chy	F	T(A/T)GTAGCCCA(T/C)TTTCATTA(T/C)GT	Ribera et al. 2010
	Tom	R	AC(A/G)TAATGAAA(A/G)TGGGCTAC(T/A)A	Ribera et al. 2010
*rrnL*–*nad1*	16saR (M14)	F	CGCCTGTTTA(A/T)CAAAAACAT	Simon et al. 1994
	16Sa	R	ATGTTTTTGTTAAACAGGCG	Simon et al. 1994
	16Sb	R	CCGGTCTGAACTCAGATCATGT	Simon et al. 1994
	ND1A (M223)	R	GGTCCCTTACGAATTTGAATATATCCT	Simon et al. 1994
*LSU*	D1	F	GGGAGGAAAAGAAACTAAC	Ober 2002
	D3	R	GCATAGTTCACCATCTTTC	Ober 2002

### Phylogenetic analyses

We aligned the sequences using the MAFFT online v.6 and the Q–INS–i algorithm (Katoh and Toh 2008), a progressive pair–wise method with secondary refinement. We used Maximum Likelihood as implemented in the on–line version of RAxML (which includes an estimation of bootstrap node support, Stamatakis et al. 2008), using GTR+G as the evolutionary model and three partitions corresponding to the *cox1*, *rrnL*+*trnL*+*nad1* and *LSU* fragments.

The aedeagus and genital duct were extracted and included in a drop of Canada balsam or dimethyl hydantoin formaldehyde resin (DMHF) on a transparent slide. Preparations were mounted below the specimen, on the same pin. Pictures were taken with microscopes Olympus ch and Olympus szx16, coupled with a camera Olympus c5060wz. Serial pictures were combined using the CombineZP software, and finally processed using Adobe Photoshop CS.

### Institutional codes and abbreviations used in the taxonomic treatment and private collectors

IBEInstitute of Evolutionary Biology (CSIC-UPF), Barcelona (Spain).


MNCNMuseo Nacional de Ciencias Naturales (CSIC), Madrid (Spain).


MNHNMuséum National d´Histoire Naturelle, Paris (France).


MZBMuseu de Ciències Naturals (Zoologia), Barcelona (Spain).


ZSMZoologische Staatssammlung, München (Germany).


MFNMuseum für Naturkunde, Berlin (Germany).


CAFcoll. A. Faille (Paris, France).


CCBcoll. C. Bourdeau (Rebigue, France).


CJFcoll. J. Fresneda (Llesp, Spain).


CMTcoll. M. Toribio (Madrid, Spain).


LELength of elytra.


LPLength of pronotum.


WEWidth of elytra.


WHWidth of head.


WPWidth of pronotum.


WPBWidth of pronotal base.


## Results

### Trechus bouilloni

Faille, Bourdeau & Fresneda sp. n.

urn:lsid:zoobank.org:act:C967CB33-C16A-468F-B786-E6F376B2D978

http://species-id.net/wiki/Trechus_bouilloni

Figs 1 8 15, 16 29

#### Type locality.

Spain, Navarra,Sierra de Urbasa–Andía, Lizarraga, Puerto de Lizarraga, UTM (WGS 84): 30 T, X: 580, Y: 4746, Z: 900 m.

#### Type series.

Holotype (MNHN): 1 ♂, Spain, Navarra,Sierra de Urbasa–Andía, Lizarraga, Puerto de Lizarraga, MSS, trap: 1–5–1980/15–8–1980, Bourdeau and Fresneda leg., voucher number ZSM–L201, MNHN]. DNA aliquotes preserved in the DNA and tissue collections of the ZSM, MNHN and IBE; Genitalia dissected and mounted in a separate label pinned with the specimen. Paratypes: 52 ♂♂, 62 ♀♀, same label data as holotype (MNCN, MNHN, MZB, ZSM, CCB, CJF, CAF, CMT).

#### Diagnosis.

**Figure 1.**
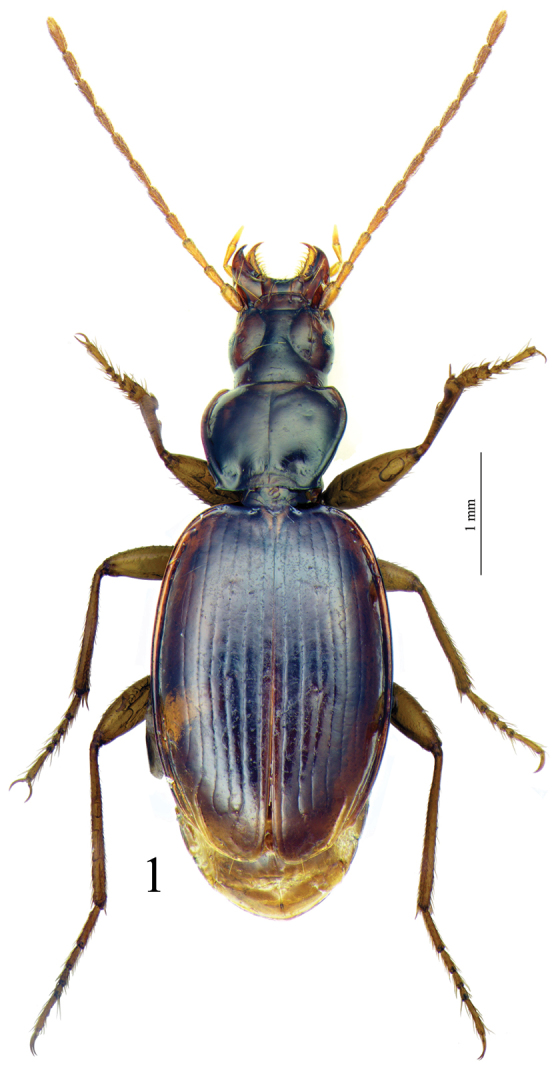
Habitus of *Trechus bouilloni* sp. n. (Lizarraga pass).

Large size (ca 5 mm) and round shape (Fig. 1). Median lobe of aedeagus slender, in lateral view (Fig. 15) the basal third curved, the central part straight and the apex with a curved hook assymetrical in dorsal view (Fig. 8). Inner sac of aedeagus (=endophallus) with an elongate and well-sclerotized piece, forming a gut and armed with internal scales. Characteristic secondary sclerotization of the sperm duct (Fig. 15: CP2) forming a kind of second copulatory piece outside base of the median lobe.

#### Description of the holotype.

Habitus as in Fig. 1.Elongated, round–sided. Body surface with a very thin, hardly visible, dense microreticulation, with more distinguishable meshes on the head.

*Colour*. Dorsal surface dark brown, moderately shiny. Antennae, palpi and legs light brown.

*Chetotaxy*. Surface of elytra glabrous with the exception of a periscutellar seta, two discal setae on the third stria, four humeral setae, four setae along lateral margin and two preapical setae. Marginal setae of pronotum present, the anterior ones located before the first third of the length. Ventral pubescence limited to one seta on each half sternite.

*Head*. Eyes reduced, flat; ommatidia well defined; maximum diameter of about eight ommatidia, temples approximately twice the length of eyes, strongly wrinkled to the neck. Frontal furrows deeply impressed. Antennae moderately long, five antennomeres extend beyond the pronotal base. Antennomere III distinctly longer than antennomeres II and IV, which are similar in length.

*Pronotum*. Proportions (M–F): WP/LP = 1.3–1.28, WP/WPB = 1.3–1.3, WP/WH = 1.38–1.3, WE/WP = 1.57–1.53. Transverse, with lateral margins finely bordered; wider in anterior part, narrower than elytra; posterior part much narrower than base of elytra. One seta in the marginal gutter at about a third of pronotum length, another one close to hind angle. Sides evenly rounded and straight just between hind angles and insertions of posterior setae. Hind angles well developed, salient.

*Elytra*. Proportions (M–F): WE/LE = 0.65–0.69. Oval, broadest almost at mid–length; surface moderately convex, flattened on disc. Shoulders distinct but rounded. Striae very finely punctuated, sixth inner striae deeply impressed on disc, but reduced at apex and base; seventh striae shallower, but distinct, the eighth reduced to the posterior quarter of elytra. Apical striola strongly impressed continuing the fifth stria.

*Hind wings*. Very reduced, not functional.

*Male genitalia*. Median lobe of aedeagus slender, in lateral view (Fig. 15) the basal third curved, the central part straight and the apex showing a curved hook; assymetrical in dorsal view (Fig. 8). Parameres slender, each with 4 to 6 setae at apex. Internal sac of aedeagus with an elongate well-sclerotized piece, forming a symmetrical gut and armed with internal scales (Fig. 16). Characteristic secondary sclerotization of the sperm duct forming a kind of second copulatory piece out of the base of the median lobe (Fig. 15: CP2).

#### Female genitalia.

Internal genitalia membranous. Gonocoxites unguiform, with 4 to 5 large setae, and 2 small near apex. Gonosubcoxites with 2 to 3 large setae near the internal edge. Laterotergite IX with 12 setae at the basal margin, and 4 to 6 scattered (Fig. 29).

#### Size.

Mean length (5 exemplars): 5.25 mm (male), 4.56 mm (female).

#### Etymology.

The new species is dedicated to Michel Bouillon, Pyrenean speleologist, who was the first to discover the existence of cave beetles in MSS.

#### Affinities.

*Trechus bouilloni* sp. n. is a representative of the *Trechus brucki* group sensu novo as defined in the present paper. It shares with *Trechus grenieri*, *Trechus uhagoni*, *Trechus beusti*, and *Trechus pieltaini* the same kind of aedeagus morphology, especially the apex with a curved hook in lateral view, and an internal sac showing two sclerotized parts, the internal copulatory piece and another triangular piece forming a kind of second copulatory piece (CP2, Figs 17–24), also existing in *Trechus brucki* and *Trechus bruckoides* sp. n. (Figs 25–28). Similar secondary sclerotized structures of endophallus are known in some groups of insects including Coleoptera, and described as a “sperm pump” (Beutel and Leschen 2005, Hünefeld and Beutel 2005, Jäch and Delgado 2010). In the *Trechus brucki* group, however, the structure is too rudimentary to play the same role in sperm transfer, and its function –if any– remains obscure. Although never observed before, this structure is also present in the others species of the group and is the main synapomorphy of the clade. The lack of this internal sclerotized structure in *Trechus carrilloi* and *Trechus sharpi* (Figs 30–32) casts doubt on their affinities.

#### Distribution and ecology.

*Trechus bouilloni* sp. n. is only known from the type locality, the MSS of Lizarraga pass (Navarra, Spain) (Fig. 36). The type locality is a MSS located on a northern slope at the eastern extremity of the Sierra de Andía–Urbasa, close to the Lizarraga pass.

*Trechus* were collected by means of traps in a zone of scree (altitude: 900 m) extending from east to west at the feet of cliffs of Albian limestone lining the northern slope of the plateau of the Sierra de Andía–Urbasa. This scree slope consists of a mass of fallen rocks resulting from the erosion of calcareous cliffs and constitutes a steeply sloped (45°) MSS, filling one of the numerous gullies of a beech forest covering the entire northern side of the plateau lining the southward depression of the Río Arakil (Sakana valley).

On this unstable ground, beeches are replaced by grassy and mossy vegetation dotted with shrubs. The layer of humus is irregular and very thin and only partly covers the blocks of white, angular, medium–sized limestone, rarely exceeding the size of 1 dm³.

The traps were placed 50 centimeters deep in a “C–type” horizon (sensu Juberthie et al. 1981), constituted mainly by stones of 5 cm³, not sealed by the ground and not welded, leaving numerous spaces between them and forming a layer several meters thick above the compact rock.

The other Coleoptera collected with *Trechus bouilloni* sp. n. were Leiodidae, Cholevinae: *Catops subfuscus* Kellner, 1846, *Sciodrepoides watsoni* (Spence, 1813) (Catopini) and *Bathysciola* sp. (Leptodirini).

Some specimens of *Trechus bouilloni* sp. n. were parasitized by an undetermined Ascomycete.

*Trechus bouilloni* sp. n. was not found in caves of the area north of Larraona (cueva de los Cristinos, cuevas de Erbeltz, Txintxoleze, Noriturri, Akuandi, del Queso, Iniriturri, Arleze, Laminatitur), suggesting that it is strictly located in MSS (CB personal observation).

### Trechus brucki

Fairmaire, 1862

http://species-id.net/wiki/Trechus_brucki

Figs 7 14 27, 28 33

#### Type locality.

**«**Eaux–Bonnes, M. vom Bruck» (Fairmaire, 1862b). France, Pyrénées–Atlantiques.

#### Type series.

Lectotype (MNHN), present designation: 1 ♂, labelled: «oblongulus Bonnes» [white rectangular label (ms, Fairmaire)], «Bruckii» [white rectangular label (ms, Fairmaire)], «MUSEUM PARIS Collection Léon Fairmaire 1906» [white rectangular label (printed)], «TYPE» [red rectangular label (printed)], «Lectotypus / *Trechus bruckii* Fairmaire / Faille, Bourdeau & / Fresneda des. 2012” [red rectangular label (printed)], genitalia dissected and mounted in a separate label pinned with the specimen. Paralectotype (MNHN): 1 ♀, same label data and pin as lectotype except “Paralectotypus / *Trechus bruckii* Fairmaire / Faille, Bourdeau & / Fresneda des. 2012” [red rectangular label (printed)].

#### Type series of *Trechus planiusculus* Fairmaire, 1862.

Lectotype (MNHN), present designation: 1 ♀ (red dot), labelled: “oblongus” [white rectangular label (ms, Fairmaire)], “planiusculus” [white rectangular label (ms, Fairmaire)], “Bruckii” [white rectangular label (ms, Fairmaire)], “2203” [white rectangular label (ms, Fairmaire)], “MUSEUM PARIS Collection Léon Fairmaire 1906” [white rectangular label (printed)], “TYPE” [red rectangular label (printed)], “Lectotypus / *Trechus planiusculus* Fairmaire / Faille, Bourdeau & / Fresneda des. 2012” [red rectangular label (printed)]. Paralectotypes (MNHN): 1 ♀, same label data and pin as lectotype except «Paralectotypus / *Trechus planiusculus* Frm / Faille, Bourdeau & / Fresneda des. 2012” [red rectangular label (printed)]; 1 ♂, “H Pyrenees 1856 M. Pandellé” [white rectangular label (printed)], “Bruckii” [white rectangular label (ms, Fairmaire)], “COTYPE” [white and red rectangular label (printed)], “R. Jeannel Brucki Fr” [white rectangular label (ms, Jeannel)], “MUSEUM PARIS coll. R. JEANNEL 1931” [white rectangular label (printed)], “Paralectotypus / *Trechus planiusculus* Frm / Faille, Bourdeau & / Fresneda des. 2012” [red rectangular label (printed)], genitalia dissected and mounted in a separate label pinned with the specimen.

#### Non Type material.

1 ♀ (MNHN) labelled: “planiusculus” [white rectangular label (ms, Fairmaire ?)], “Bruckii” [white rectangular label (ms, Fairmaire)], “MUSEUM PARIS Collection Léon Fairmaire 1906” [white rectangular label (printed)], “R. Jeannel Brucki Fr” [white rectangular label (ms, Jeannel)]. We do not consider this specimen as a syntype of *Trechus planiusculus* as it is not labeled « oblongus » as the specimen of the type series, suggesting that the specimen arrived in the Fairmaire collection after the description of *planiusculus*. A second female specimen (MNHN) labelled: “oblongus Arrens” [white rectangular label (ms, Fairmaire)], “TYPE” [white and red rectangular label (printed)], “MUSEUM PARIS Collection Léon Fairmaire 1906” [white rectangular label (printed)]. This specimen could be the reference specimen of *Trechus oblongus* Schaum, 1862. Reference of the name comes from Schaum (1862: addenda, p. 119): “P. 14 col. 2 *Trechus oblongus* Schaum;” only the name is mentioned, without any description, number of exemplars studied or locality. It should then be considered as nomen nudum. Jeannel (1927) indicates that *Trechus oblongus* is a synonym of *Trechus brucki* with type locality: “Pyrén. occ.” We were unable to find the specimen or reference where Jeannel found the type locality.

##### Taxonomic comments

The study of specimens of *Trechus brucki pecoudi* from Orhy and of numerous exemplars of *Trechus brucki*,including types of the previously described subspecies of *Trechus brucki*,demonstrated that none of the characters quoted either by Colas and Gaudin (1935) or by Queinnec and Ollivier (2011) are constant. We consider then the subspecies *pecoudi* as synonymous of *Trechus brucki*: *Trechus brucki brucki* Fairmaire, 1862 = *Trechus brucki pecoudi* Colas & Gaudin, 1935, syn. n.

*Trechus politus* and *Trechus planiusculus* were described by Fairmaire in the volume of the Annales de la Société Entomologique de France of 1861 published in 1862 (Fairmaire, 1862b). As the name *Trechus politus* was already used for an American species (today *Trechisibus politus* Brullé, 1842), Fairmaire (1862a) changed the name of this species to *Trechus bruckii*. *Trechus planiusculus* was considered synonymous with *Trechus brucki* by Jeannel (1927), and, moreover, the name *planiusculus* was preoccupied as it was used by Costa (1858) in a work on Italian fauna. In his works on Trechini, Jeannel (1927, 1941) illustrated the genitalia of a male from the Ossau Valley. Recently, in a revision of the french fauna of Carabidae, Queinnec and Ollivier (2011) suggested that the drawing of Jeannel (1927, 1941) was incorrect, and that the male genitalia of *Trechus brucki* has a homogeneous shape throughout the distribution area. By examining the types of Fairmaire, we noticed that the drawing of Jeannel (1927, 1941) does not actually match with *Trechus brucki*. However, by studying specimens from Ossau Valley we found that the drawing of Jeannel actually corresponds to another undescribed species, very narrowly located in the area of Pic de Montagnon (Bielle–Pyrénées Atlantiques). Here we describe this new species as *Trechus bruckoides* sp. n.

### Trechus bruckoides

Faille, Bourdeau & Fresneda sp. n.

urn:lsid:zoobank.org:act:030DC2D3-4509-4877-8C21-9D83AA8563B5

http://species-id.net/wiki/Trechus_bruckoides

Figs 6 13 25, 26

#### Type locality.

France, Pyrénées Atlantiques, Ossau, Sède de Pan UTM (WGS 84): 30 T, X:704, Y:4768.

#### Type series.

Holotype (MNHN): 1 ♂, France, Pyrénées Atlantiques, Ossau, Sède de Pan, labelled: «Ossau, Sède–Pan» [white rectangular label (printed)], «MUSEUM PARIS coll. R. JEANNEL 1931» [white rectangular label (printed)], «R. Jeannel Brucki Fr.» [white rectangular label (ms, Jeannel)], «Holotypus / *Trechus bruckoides* sp. n. / Faille, Bourdeau & / Fresneda det. 2012” [red rectangular label (printed)], genitalia dissected and mounted in a separate label pinned with the specimen. Paratypes: 1 ♂, “Pic Montagnoü (v. d´Ossau) Mascaraux” [white rectangular label (ms)], “MUSEUM PARIS 1932 coll. Sainte–Claire Deville” [white rectangular label (printed)], “angusticollis Kiesw.” [white rectangular label (ms)] (MNHN); 1 ♂, “Pic Massibe B. PYR. 1938” [white rectangular label (ms)], “Trechus Brucki” [white rectangular label (ms)], “Collection H. Coiffait” [white rectangular label (printed)] (MNHN); 1 ♂, “Bielle/ B. Pyr.” “Trechus brucki/det. Tedeschi” “coll. Tedeschi/ZSM 2009” (ZSM); Pic Montagnon, 15–VII–1979, Bourdeau leg., 6 ♂♂ and 1 ♀ (CAF, CCB, CJF); Sède de Pan, Bielle, VII–1995, Bourdeau leg., 1 ♂ (CCB); Sède de Pan, Bielle, 2–VIII–1980, Bourdeau leg., 3 ♂♂ (CCB); Sède de Pan, Bielle, 10–VII–1981, Bourdeau leg., 1 ♂ and 2 ♀♀ (CCB). All the paratypes with the label “Paratypus / *Trechus bruckoides* sp. n. / Faille, Bourdeau & / Fresneda det. 2012” [red rectangular label (printed)].

#### Supplementary specimen studied. 

1 ♀, «Pied du pic Lauriolle près Bielle Bas. Pyr. 29.6.37», coll. Bonnaire (MNHN). Sède de Pan, Mascaraux, 2 exx. (coll. Nègre, MNHN). Pic Montagnon: 4 exx. Sède de Pan: 3 exx (MNHN). Sède de Pan, 23–6–1943, 1 ♂, 2 ♀♀ (MNHN, coll. Coiffait). Pic Massibe: VII–1941, 1 ♀ (MNHN, coll.Coiffait).

#### Diagnosis.

Large size (ca 4 mm) and round shape (Fig. 6). Median lobe of aedeagus slender, subparallel and decreasing in width from the apical tenth to the apex, which is softly curved in lateral view (Fig. 25), nearly symmetrical and with apex regularly rounded in dorsal view (Fig. 13). Endophallus with an elongate and well-sclerotized piece, forming a twisted gut. Characteristic secondary sclerotization of the sperm duct (Fig 25: CP2) present. External appearance very close to *Trechus brucki*.

#### Description of the holotype.

Habitus as in Fig. 6.Elongated, round–sided. Body surface with a very thin, hardly visible, dense microreticulation, no more distinguishable meshes on the head.

*Colour*. Dorsal surface dark brown, moderately shiny. Antennae, palpi and legs light brown.

*Chetotaxy*. Surface of elytra glabrous with the exception of a periscutellar seta, two discal setae on the third stria, four humeral setae, four setae along lateral margin and two preapical setae. Marginal setae of pronotum present, the anterior ones located at the first anterior third of the length.

*Head*. Eyes flat, well–developed, temples smaller than the length of eyes, strongly wrinkled to the neck. Frontal furrows moderately deep. Antennae short (2–2.3mm) and thick.

*Pronotum*. Proportions (M): WP/LP = 1.3, WP/WPB = 1.35, WP/WH = 1.34, WE/WP = 1.63. Transverse, with lateral margins bordered, wider in anterior part, much less wide than elytra. Posterior part much narrower than base of elytra. One seta in the marginal gutter at about a third of pronotum length, another one just before hind angle. Sides evenly rounded and straight just between hind angles and insertions of posterior setae. Hind angles well developed, right.

*Elytra*. Proportions (M): WE/LE = 0.64. Subrectangular, broadest after the mid–length; surface moderately convex, flattened on disc. Shoulders distinct but rounded. Striae almost impunctuate, sixth inner discal striae distinct, but reduced at apex and base, especially in callus area; seventh striae shallower, nearly indistinct, the eighth only distinct close to apex of elytra. Apical striola well impressed continuing the fifth stria.

*Hind wings*. Very reduced, not functional.

*Male genitalia*. Median lobe of aedeagus slender, in lateral view (Fig 25) basal third curved, central part straight, parallel and elongated towards apex. Nearly symmetrical in dorsal view (Fig 13). Parameres slender, each with 4 setae at tip. Inner sac of aedeagus armed with scales with an elongate well sclerotized piece, forming a twisted gut (Fig 26). Characteristic secondary sclerotization of the sperm duct forming a kind of second copulatory piece out of the base of the median lobe (Fig 25: CP2).

#### Female genitalia.

Not examined.

#### Size.

Mean length (4 exemplars): 4.78 mm (male).

#### Etymology.

The specific epithet refers to *Trechus brucki*, species with which the new species was merged.

#### Affinities.

*Trechus brucki* and *Trechus bruckoides* sp. n. are externally very similar but strong differences isolate the two taxa especially in shape of male genitalia (Figs 25, 27). The aedeagus shape of *Trechus bruckoides* sp. n. is exactly as indicated in Jeannel (1927, 1941) for *Trechus brucki*.

#### Distribution and ecology.

*Trechus bruckoides* sp. n. is only known from the calcareous plateau of Esturou located at 1860 m, north of Montagnon peak (1973 m) and Mailh Massibé (1973 m), at the northern extremity of the massifs separating Aspe and Ossau valleys (Fig. 37). South of this area (Sesques and Gaziès peaks (2600 m)), it is replaced by *Trechus brucki* which occurs together with *Trechus distinctus*. During Pleistocene glacial cycles, this plateau was covered by a névé which shaped an area of sinks of nivo–karstic origin (Auly 2008). After winter, snow remains in these sinkholes (July–August) and allows the preservation of a nivicolous fauna, which is unusual at these medium altitudes. *Trechus bruckoides* sp. n. lives exclusively in the masses of fallen rocks of sinkholes and follows the withdrawal of the snow. When the snow thaws it likely seeks refuge underground.

This mid altitude nivicolous environment could have led to isolation of populations of the species from southern glaciated areas and glacial tongues of the northern slope of Ossau glacier and led to the differentiation of this population of cryophilic and highly hygrophilic *Trechus*. Such a hypothesis could also explain the presence of the hypogean Trechini *Aphaenops bessoni* Cabidoche, 1962, endemic to this karstic plateau (pits of Col d’Aran), and closely related to *Aphaenops loubensi* Jeannel, 1953, an endemic species of the Pierre Saint Martin massif, western to the Aspe Valley. Some other endemic nivicolous Carabidae with morphologically distinct populations occur in the area, like *Carabus* (*Iniopachus) pyrenaeus* Audinet–Serville, 1821 (the population of Sède de Pan was first described as a distinct subspecies, *Carabus pyrenaeus cephalicus* Csiki, 1927), *Nebria lafresnayei* Audinet–Serville, 1821, *Pterostichus (Cryobius) amoenus mascarauxianus* Pupier, 2008, *Pterostichus (Lianoe) nadari mascarauxi* Jeannel, 1928 and *Pterostichus (Lianoe) dufourii* (Dejean 1828). The peculiarities of this fauna suggest that this restricted area is an important center of diversification.

## Discussion

**Figures 2–3.**
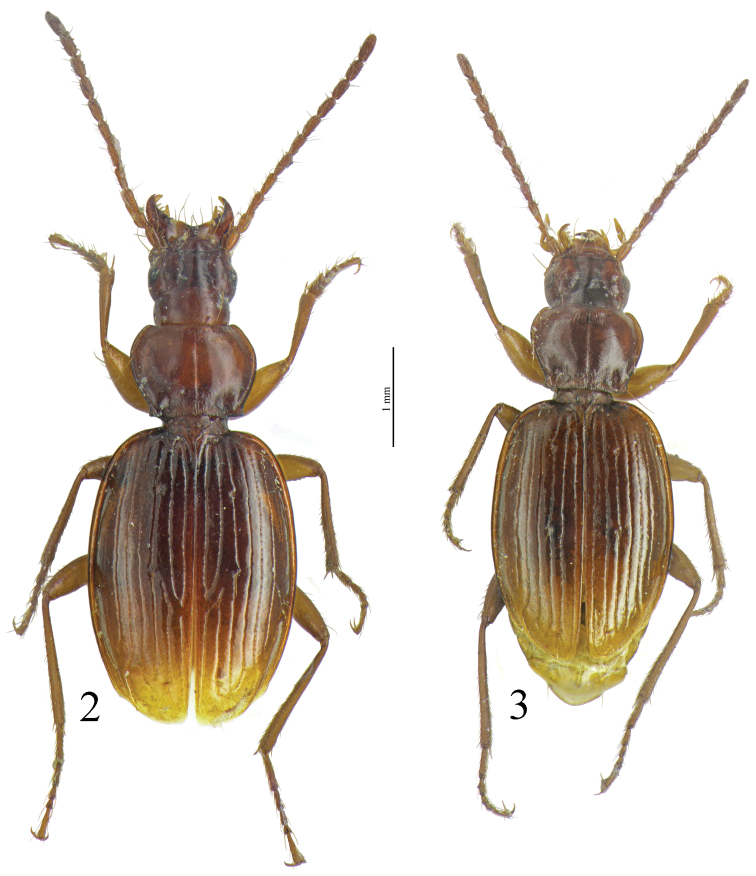
Habitus of **2** *Trechus grenieri* (grotte de l´Eglise) and **3** *Trechus uhagoni* (Orobe doline).

**Figures 4–5.**
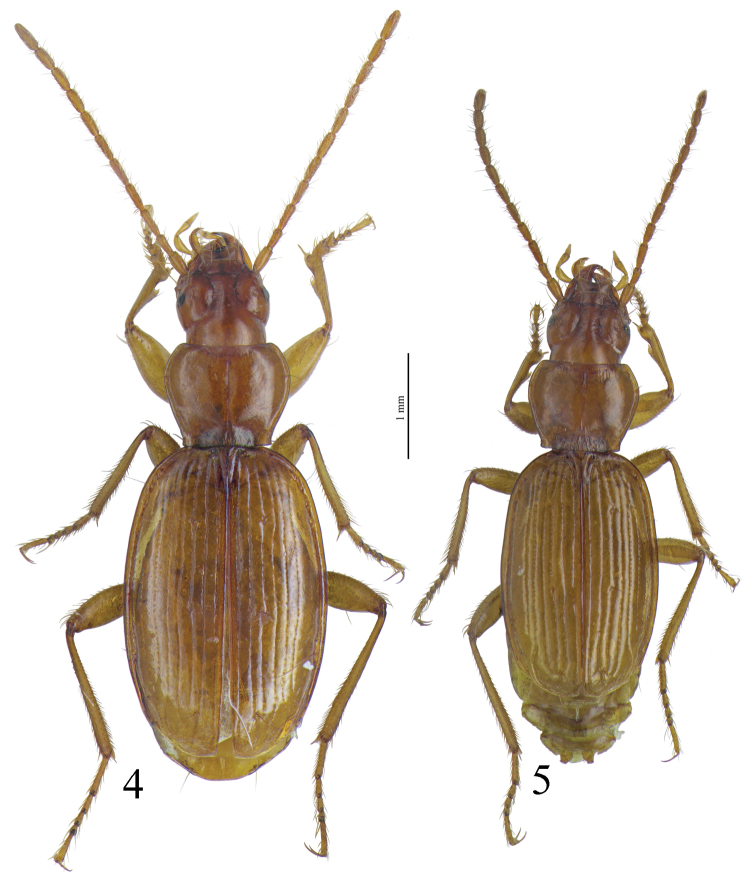
Habitus of **4** *Trechus beusti* (Cueva de San Adrián) and **5** *Trechus pieltaini* (Cueva de Mairuelegorreta).

The molecular phylogeny (Fig. 34) suggests a well–supported clade gathering the following species:

*Trechus beusti* (Fig. 4), *Trechus bouilloni* sp. n.(Fig. 1), *Trechus grenieri* (Fig. 2), *Trechus brucki* (Fig. 7), *Trechus pieltaini* (Fig. 5) and *Trechus uhagoni* (Fig. 3). This result is in accordance with morphology: all the species of the clade share the aedeagal median lobe long and strongly curved just behind basal bulb, with terminal lamella well–developed. Moreover, the clade is supported by a strong synapomorphy: all the species share a strongly sclerotized part of the sperm duct, forming a second copulatory piece (Figs 15, 17, 19, 21, 23, 27: CP2). This synapomorphy is also present in *Trechus bruckoides* sp. n.(Fig. 25). Consequently, molecular and morphological results allow us to define the *Trechus brucki* group sensu novo: *Trechus beusti*, *Trechus bouilloni* sp. n., *Trechus brucki*, *Trechus bruckoides* sp. n., *Trechus grenieri*, *Trechus uhagoni* and *Trechus pieltaini*.

Two species, *Trechus pieltaini* and *Trechus beusti*, were included by Jeannel (1927) in the *angusticollis* group. *Trechus carrilloi* and *Trechus sharpi* are provisionally not included in the group because of the absence of sclerotization of the sperm duct (CP2). Moreover, concerning *Trechus carrilloi*, the apical hook is not a synapomorphy, as several other *Trechus* groups have this kind of hook (i.e. *Trechus aubryi* or the Tibetan species *Trechus bastropi* Schmidt, 2009 and *Trechus damchungensis* Deuve, 1997). As expected by Dupré (1991) for some of the species that he included in a “*bonvouloiri* group”, the following regional species previously considered part of the *brucki* group are clearly excluded here: *Trechus escalerai*, *Trechus navaricus*, *Trechus bordei*, *Trechus jeannei*, *Trechus bonvouloiri*. Although not included in our analyses, we also exclude of the group *Trechus ortizi*, *Trechus baztanensis* and *Trechus enigmaticus*, as those species were put into the *uhagoni* group because of close morphological affinities with *Trechus bordei* and *Trechus navaricus* (Español 1970, Coiffait 1971, Dupré 1991). *Trechus jeannei*, which was said to be close to *Trechus bordei* (Sciaky 1998), does not belong to the *Trechus brucki* clade and is not clearly related with the *Trechus bordei* clade. A study including more Iberian species should clarify its phylogenetic affinities. *Trechus aubryi* is excluded from the *Trechus brucki* clade and shares strong affinities with *Trechus distinctus*.

Our molecular results as well as genital morphology, in dorsal and lateral view and shape of copulatory piece (Figs 10, 19, 20), suggest that *Trechus uhagoni* could be considered a distinct species from *Trechus grenieri*. Morphological differences between the two species are the following:

– *Trechus grenieri*: aedeagus in dorsal view (Fig. 9) with subparallel sides, round apex with a short triangular tip; in lateral view (Fig. 17) basal third strongly rounded, median lobe slightly angular in the middle; apical hook with a thin tip. The copulatory piece is an asymmetrical gut slightly tapering and filled with a densely scaly area (Fig. 18).

– *Trechus uhagoni*: aedeagus in dorsal view (Fig. 10) with the left side narrowed or sinuate from the middle to apex, the left side of apical quarter deeply narrowed, forming a long triangular tip; in lateral view (Fig. 19) only the basal quarter rounded, median lobe without dorsal angle in the middle, short with apical hook with massive tip. The copulatory piece is similar to the one of *grenieri* but the gut is parallel and shortened in its apical part (Fig. 20).

With 7 subspecies recognized in the last catalogues (Moravec et al 2003, Queinnec and Ollivier 2011), *Trechus grenieri* currently lives in humid forests (1000 m) from Espinal to Iraty (ssp. *ruteri*), then from Gave de Pau river (north side of Pic de Montaigu) to the Neste d’Aure valley (ssp. *grenieri*). From Aure valley to the Salat, it is replaced by the subspecies *despaxi*, which crosses the Garonne river near Saint Béat (Haute Garonne). An isolated subspecies (*prepyrenaeus*) was described by Coiffait in the high Arize valley (Andronne and Bosc forests around 1000 m) (Coiffait 1974). Along the axial ridge, *Trechus grenieri* lives above 1500 m from high Garonne valley (ssp. *bepmalei*) to Mont Valier (ssp. *aulaensis* Aubry, 1981). Study of numerous specimens suggests that *Trechus uhagoni* and all the specimens of the subspecies *ruteri* are morphologically close, especially in the shape of the male genitalia, and should be considered as a distinct species. Moreover, we studied specimens from various localities of the Pyrenean range (see Distribution) and established that they share some morphological characters (color pale, brown, pronotum transverse with lateral margin regularly rounded, elytral striae superficial, weakly impressed) that justify keeping the status of *ruteri* as a subspecies of *Trechus uhagoni*. The subspecies *ruteri* should then be considered as belonging to *uhagoni*, so that *grenieri* is restricted to the area between Gave de Pau and Ariège valley, northern slope of Pyrenees. *Trechus uhagoni ruteri* n. comb. could be distinguished from *Trechus uhagoni uhagoni* by its color, usually paler brown, the pronotum transverse with lateral margin regularly rounded and the elytral striae superficial, weakly impressed. It is restricted to the western Pyrenees.

The study of specimens from the whole range of *Trechus grenieri* including all the subspecies, most of the types and material from intermediate localities (see distribution) leads us to conclude that the characters used to discriminate the subspecies (size, eyes size, shape of elytra and pronotum) are inconstant and overlapping between populations. The shape of the male genitalia is similar for all the populations between Gave de Pau and Ariège valley, including the one (ssp *aulaensis*) which was said to be different (Queinnec and Ollivier 2011). We consider then *Trechus grenieri* as a single species without any valid subspecies: *Trechus grenieri grenieri* Pandellé, 1867 = *Trechus grenieri bepmalei* Jeannel, 1921 = *Trechus grenieri despaxi* Jeannel, 1922 = *Trechus grenieri aulaensis* Aubry, 1981 = *Trechus grenieri prepyrenaeus* Coiffait, 1974,syn.n.

*Trechus beusti* was described by Schaufuss (1863). The type series is located in the Schaufuss collection in the Museum für Naturkunde, Berlin (M. Jaeger pers. com.). *Trechus pieltaini* was described by Jeannel (1920) from a cave of the Basque country, Cueva de Mairruelegorreta. Bolívar y Pieltain and Jeannel (1921) suggested that the peculiar morphology of the aedeagus of these two species indicates clear affinities with *Trechus uhagoni* and *Trechus grenieri*. Surprisingly, Jeannel (1927) in his Monographie des Trechinae considered that these two species belong to another group of species, the *Trechus angusticollis* group. This opinion was followed by subsequent authors (Español 1965, Casale and Laneyrie 1982, Ortuño and Marcos 2003). However, and in accordance with the morphology of the median lobe of the aedeagus, molecular results support Bolívar y Pieltain and Jeannel’s (1921) point of view and confirm the close affinities between *Trechus beusti*, *Trechus pieltaini* and thespecies of the *Trechus brucki* clade. Differences between the two species are weak: the apical part of the aedeagus is longer in *Trechus pieltaini* (Fig. 12, 23) than in *Trechus beusti* (Fig. 11, 21). The copulatory pieces are almost identical (Fig. 22, 24). *Trechus beusti* is larger, with elytra more oval and elytral striae less impressed (Fig. 4); *Trechus pieltaini* is smaller, narrower, and elongate with subparallel elytra and striae more impressed (Fig. 5).

*Trechus brucki* and *Trechus bruckoides* sp. n.do not have the peculiar hooked apex of the median lobe observed in the other species of the clade, but the apex is nevertheless strongly curved (Figs 25, 27).

The case of two further species remains doubtful: *Trechus carrilloi* was included by its descriptor in the *uhagoni* group especially because of the structure of the aedeagus, with an apex with an apical hook (Fig. 31). However, the secondary sclerotization of the ejaculatory duct is lacking in this species and it is characterized by a homogenous elytral pubescence which is present in other species of the area (Ortuño and Jimenez–Valverde 2011), but lacking in all the species of the *Trechus brucki* clade sensu novo. The presence of a hook at the apex of the aedeagus is also known in other Pyreneo–Cantabrian species like *Trechus arribasi* Jeanne, 1988, currently included in the *Trechus fulvus* group (Toribio 2001, Reboleira et al. 2010) or *Trechus aubryi* from Ariège. This character led its descriptor to include *Trechus aubryi* in the *Trechus uhagoni* group. Queinnec and Ollivier (2011) included the species in the *Trechus angusticollis* group. The species appears to be the sister species of *Trechus distinctus* (Fig. 34).

Finally, *Trechus sharpi* was included in the *Trechus uhagoni* group by Jeannel (1927), but the shape of the median lobe of the aedeagus and the copulatory piece, that shares some similarities with the *Trechus bordei* group, together with the lack of the sclerotization of spermiduct present in all the species of the *Trechus brucki* group sensu novo, cast doubts on its real phylogenetic affinities.

The species of the *Trechus brucki* clade are humicolous (*grenieri, uhagoni*), orophilous (*grenieri*, *brucki*, *bruckoides*sp. n.), or troglobitic/subterranean (*bouilloni* sp. n., *pieltaini*, *beusti*). We can notice a coincidence in the ecology of Trechini and Leptodirini with Basque–Pyrenean distribution: whereas the species are humicolous (or nivicolous for some Trechini) in the Pyrenees, the species occurring in the Basque country are mainly hypogean (Salgado et al. 2008, Ribera et al. 2010).

### Biogeography of the *Trechus brucki* clade

If we use the standard mitochondrial mutation rate for insects of 2.3% divergence per Myr (0.0115 substitutions ⁄ site ⁄ Myr) (Brower 1994, Papadopoulou et al. 2010, Pons et al. 2010, Ribera et al. 2010), the isolation between the *Trechus navaricus* and *Trechus brucki* group seems to have occurred at the end of the Pliocene (Faille et al. 2011a). Pliocene climate was much warmer than the Present (Uriarte 2003): the interval between 3.3 Myr to 3 Myr was called *Mid Pliocene warm Period*, with an average temperature of about 3°C higher than at present and an annual average precipitation between 400 to 1000 mm higher than present. The transition to the Pleistocene (ca 2.7 Myr) is marked by the onset of marked climatic variability; the radiation of the *Trechus brucki* clade occured during the Pleistocene, following the rhythm of alternations of cold, warm/humid and dry periods that led to changes in biome composition (Salzmann et al. 2008).

Strong erosion leading to a deep excavation of Pyrenean valleys associated with climate variations led to the dispersal and diversification of the *brucki* clade. The main events are (Barrère 1963, Campos 1979, Serrat and Ventura 1993, Calvet 2004):

1. *Persistence of the Ebro depression between the Basque–Pyrenean area and the Iberian central plateau*. The persistence of the Ebro salty basin from the late Oligocene (25 Ma) until the late Miocene (6 Ma) isolated groups with an Iberian distribution from those with a Pyrenean or Basque–Pyrenean distribution. This flat and shallow lagoon area received the tributaries of the Ebro river, from Reinosa to the Mediterranean Sea.

2. *Impact of Quaternary erosion on karst fragmentation*. On the northern slope, the folds which have an east–west orientation are narrow and divided by north–south valleys. On the southern slope, orogenesis caused the formation of two folds with an east–west orientation (internal and external “sierras”) parallel to the axial chain. Similarly, Quaternary erosion separated these sierras by narrow north–south valleys. Near the Atlantic, these “sierras” meet with Basque folds which have a complex north–west/south–east orientation, divided by narrow north–south valleys, from Bilbao to Alsasua. Between Vitoria and Pamplona, these Basque “sierras” are separated by the Pre–pyrenean middle depression, a broad valley excavated by the Zadorra (westward) and Arakil (eastward) rivers (Fig 35). These rivers flow into the Ebro Basin, separating the northern massifs of Aralar, Urquilla and Gorbea from the southern Sierra of Urbasa–Andía. The hydrographic system was set mainly by significant erosion due to numerous glaciation cycles during the Pleistocene (2.5 Ma).

Our molecular study suggests that the *brucki* lineage could have originated in the area delimited by the northern sierras of Gorbea and Urquilla and the edge of the sierra de Andía. The sierras de Andía, Urbasa and Entzia form the exact border between the hypogean fauna of the Pyrenees and Iberia. North of this limit occur *Trechus bouilloni* sp. n., *Troglorites breuili* Jeannel, 1919 (Carabidae, Pterostichini) –Urbasa–Andía–Entzia, Aralar, Ernio and Pagoeta massifs, between the Deba and Urola rivers (Ortuño et al. 2010)–, *Euryspeonomus eloseguii* (Español, 1948),* Bathysciola rugosa* (Sharp, 1873), –Leiodidae, Cholevinae, Leptodirini which also belong to a clade of Basque–Pyrenean distribution (Ribera et al. 2010)– whereas south of this area (Sierras de la Demanda and Lóquiz, surrounding the Ebro basin) is characterized by a lack of Leptodirini and *Troglorites*. The only cave Coleoptera is *Trechus schaufussi comasi* (Basaula cave in Baríndano, south of Urbasa). *Trechus schaufussi* Putzeys, 1870 is a model of Iberian extensive distribution: it is widespread in the Iberian Peninsula, from Algarve in Portugal to Cantabria, Iberian Central System and the pre–pyrenean massif of Guara in Spain (Jeanne and Zaballos 1986, Zaballos and Jeanne 1994, Serrano 2003). This species is known to have separated early from *Trechus* sensu stricto (Faille et al. 2010a, 2011a).

*Trechus bouilloni* sp. n. has a subterranean lifestyle among the scree–covered northern slope (900 m) of Sierra de Andía, whereas the type locality of *Trechus uhagoni* is the Orobe doline (700 m), located at the eastern limit of Sierra de Urquilla. Early Pleistocene climate variations could have led to drastic changes in biome composition, limiting dispersal possibilities and leading to the isolation of the population of *Trechus bouilloni* sp. n. (potentially forestal), south of the Arakil River. One hypothesis could be that the hygrophilous species were colonizing high altitude or hypogean habitats during interglaciar warming as observed in other species of Coleoptera (Hernando et al. 1999, Faille et al. 2011b). These climate fluctuations might also have led to the western subterranean colonization of the two hypogean species, *Trechus beusti* (Sierra de Urquilla) and *Trechus pieltaini* (Sierra de Gorbea) while the group colonized the Pyrenean chain and diversified in numerous forms living in humid forests and alpine zones, from the Iraty Valley to the Ariège Basin. Migration toward East could have been possible along the small sierras of Tajonar and Labia, which link the Basque Mountains to the Pyrenees.

*Trechus brucki* lives in the alpine zone (above 1700 m) of the axial ridge from Pic d´Orhy to Col du Pourtalet, in the high Ossau Valley. On the north ridge, *Trechus brucki* can be encountered in the same biotopes, near snow tongues melting on scree-covered slopes, from Aspe to Gave de Pau Valleys. As for *Trechus grenieri* and *Trechus uhagoni*, both are mainly forestal and occur at lower altitude except for *Trechus grenieri* in the eastern part of the range (Mount Valier area). The Ariège Valley is the eastern limit of the group.

The distribution area of the *Trechus brucki* group coincides with the one of the Basque–Pyrenean Leptodirini clade (Fig 35). In the Pyrenees, both groups are made up of forestal, endogean, humicolous, lapidicolous or orophilous, but not hypogean, species. It is only in Basque relief, the western part of their distribution, that both groups include subterranean species. Regarding Leptodirini, the basal group of the Basque–Pyrenean clade is the *Bathysciola schiodtei* group (endogean/humicolous elements); its distribution area is extended from Ariège, *Trechus mystica* Fresneda & Fery, 2009 (France: Haute–Garonne and Ariège; Spain: Val d’Aran) to the Basque relief, *Bathysciola breuili* Bolívar, 1921 in Peña Gorbea or *Bathysciola rugosa* (Sharp, 1873) in Sierra de Urbasa and Urquilla. A high degree of troglobiomorphy is only found in some hypogean species of the Basque area: *Aranzadiella* Español, 1972 (basin of Deba River), *Euryspeonomus* Jeannel, 1919 (Aralar, Urbasa/Andía and Baztan Valley), *Josettekia* Bellés & Déliot, 1983 (Ernio and Aralar massifs), *Nafarroa* Fresneda & Dupré, 2010 (Kintoa Massif) and *Speocharidius* Jeannel, 1919 (between the Urola and Orio Rivers). In the Pyrenees, the species of the *Trechus brucki* clade are epigean, forestal (*Trechus grenieri*, *Trechus uhagoni ruteri*) or orophilous(*Trechus brucki*, *Trechus bruckoides* sp. n.). Pyrenean speciation events in the group are more recent and are probably closely related to late Pleistocene climatic changes, as already observed in alpine *Trechus* (Lohse et al. 2011). Troglobiomorphic features (depigmentation, microphthalmy) only occur in the hypogean *Trechus beusti* and *Trechus pieltaini*, both of them located in the Basque area. The two other species of this geographical area are located in wet and cold dolines (*Trechus uhagoni*) or subterranean environments (*Trechus bouilloni* sp. n.). Their general appearance (pigmented, well–developed eyes), similar to other epigean species, could be an indication of the recent colonization of this reduced habitat.

## Plates

**Figures 6–7.**
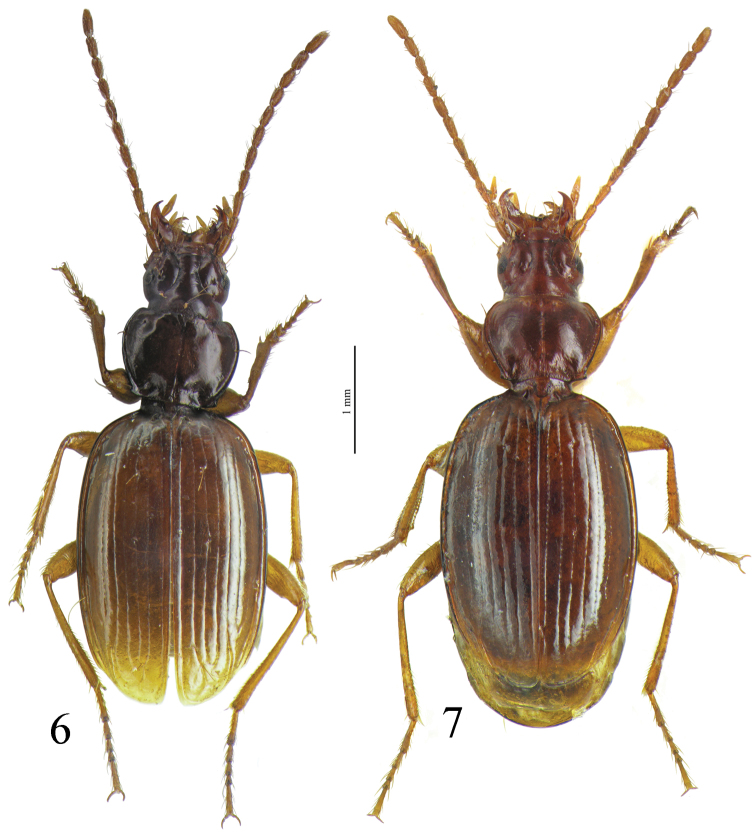
Habitus of **6** *Trechus bruckoides* sp. n. (Montagnon) and **7** *Trechus brucki* (Jaout).

**Figure 8–10.**
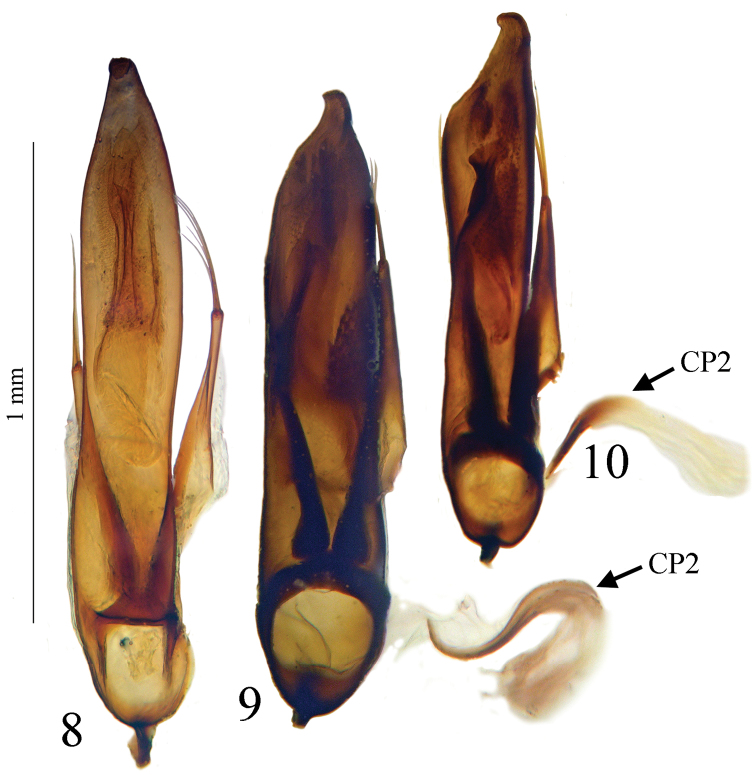
Aedeagus in dorsal view of **8** *Trechus bouilloni* sp. n. (Lizarraga pass) **9** *Trechus grenieri* (Lapiaz de Lazur) and **10** *Trechus uhagoni* (Orobe doline). CP2, secondary copulatory piece.

**Figures 11–14.**
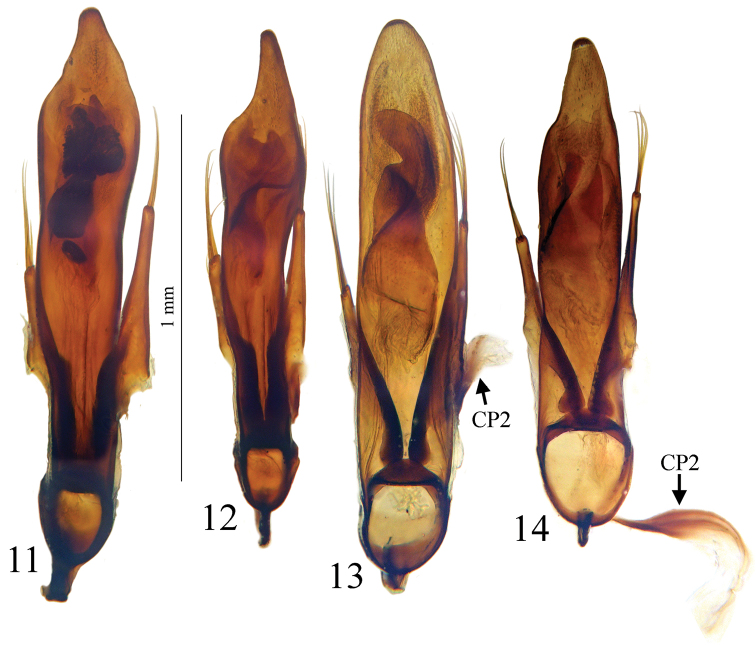
Aedeagus in dorsal view of **11** *Trechus beusti* (Cueva de San Adrián) **12** *Trechus pieltaini* (Cueva de Mairuelegorreta) **13** *Trechus bruckoides* sp. n. (Montagnon) and **14** *Trechus brucki* (Lac d’Anglas). CP2, secondary copulatory piece.

**Figures 15–20.**
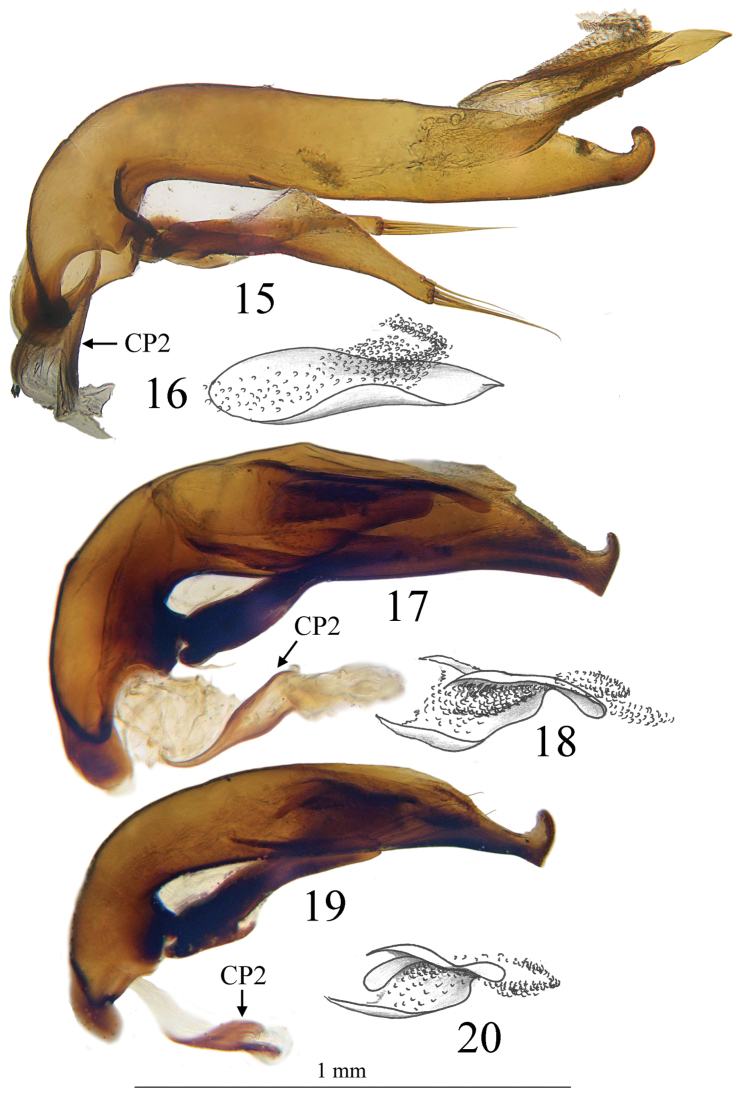
Aedeagus in lateral view and detail of internal sac of **15, 16** *Trechus bouilloni* sp. n. (Lizarraga pass) **17, 18** *Trechus grenieri* (Eglise cave) and **19, 20** *Trechus uhagoni* (Orobe doline). CP2, secondary copulatory piece.

**Figures 21–28.**
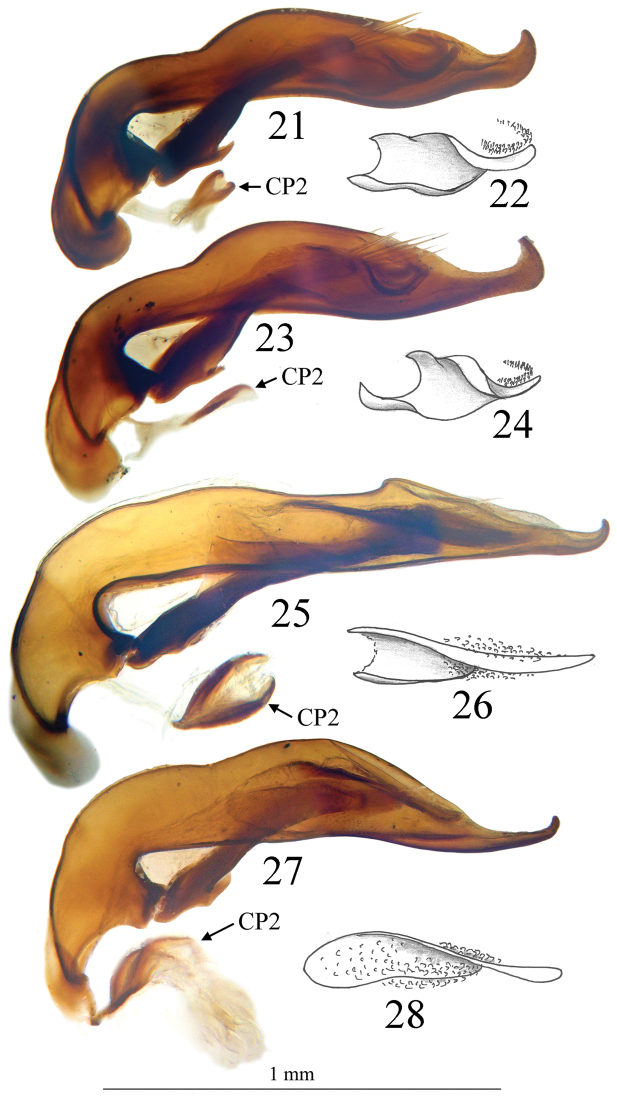
Aedeagus in lateral view and detail of internal sac of **21, 22** *Trechus beusti* (Cueva de San Adrián), **23, 24**. *Trechus pieltaini* (Cueva de Mairuelegorreta), **25, 26** *Trechus bruckoides* sp. n. (Montagnon) and **27, 28** *Trechus brucki* (Lac d’Anglas). CP2, secondary copulatory piece.

**Figures 29–32.**
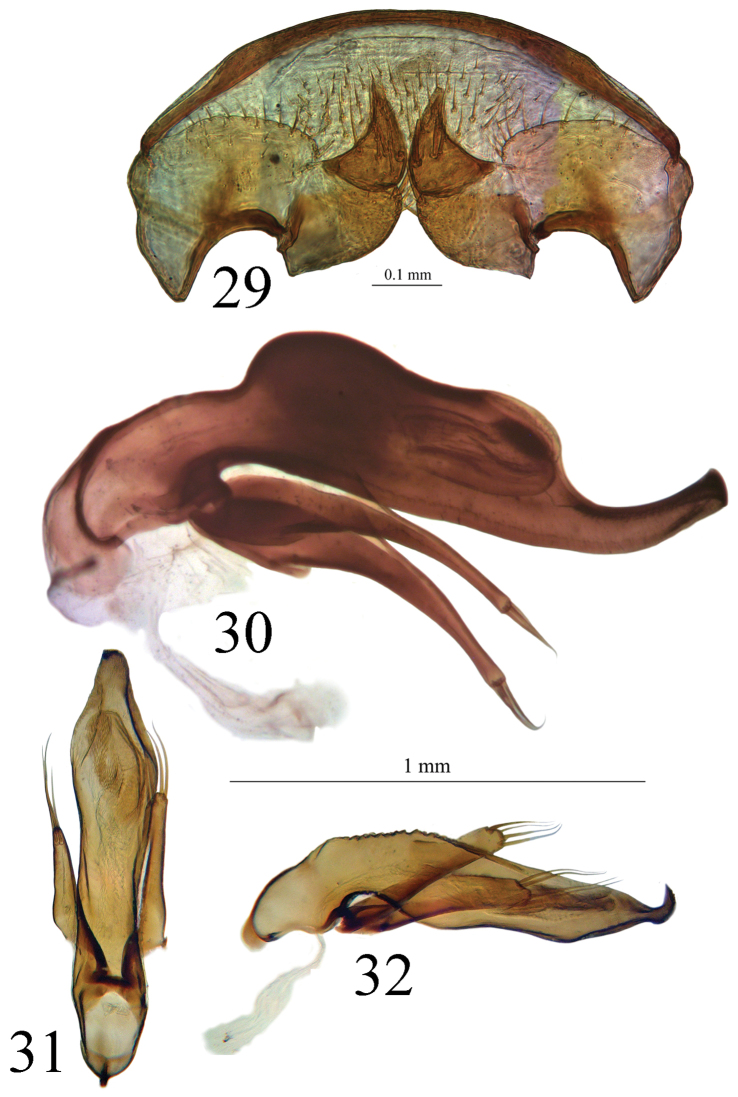
**29** Genital armature of the female of *Trechus bouilloni* sp. n. (Lizarraga pass) **30** Aedeagus in lateral view of *Trechus sharpi* (Cueva la Cuevona) **31, 32** Aedeagus in dorsal and lateral view of *Trechus carrilloi* (Bosque de Saja).

**Figure 33.**
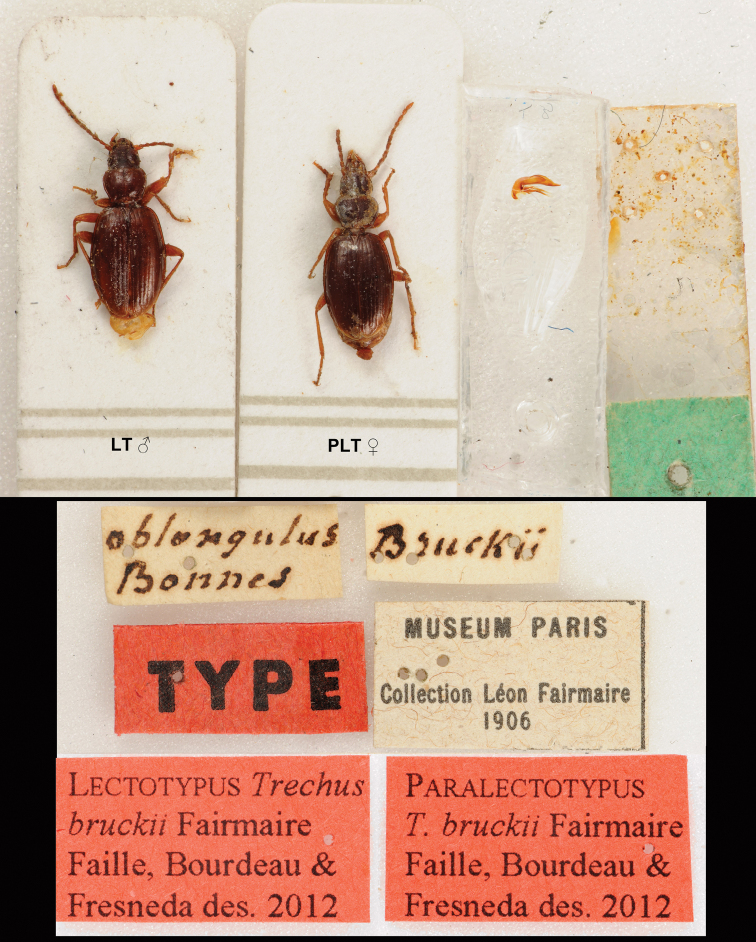
Lectotype and paralectotype of *Trechus brucki*.

**Figure 34.**
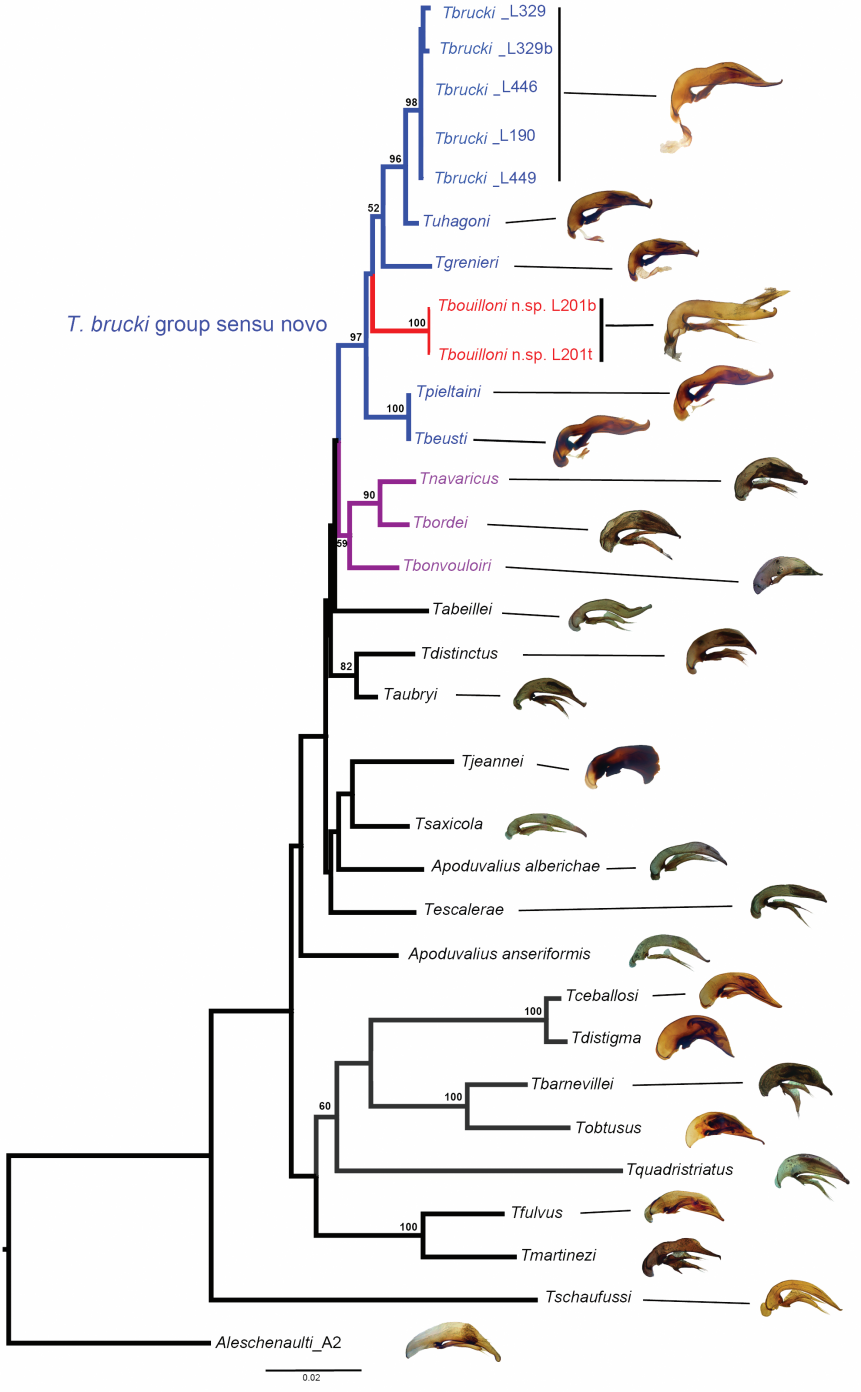
Phylogram of *Trechus* of the *brucki* group obtained in RAxML, using the combined data matrix. Number in nodes: ML bootstrap (>50%) (see Material and Methods for details). In blue, the *Trechus brucki* group sensu novo. In purple, *Trechus bordei* group. In red: *Trechus bouilloni* sp. n.

**Figure 35.**
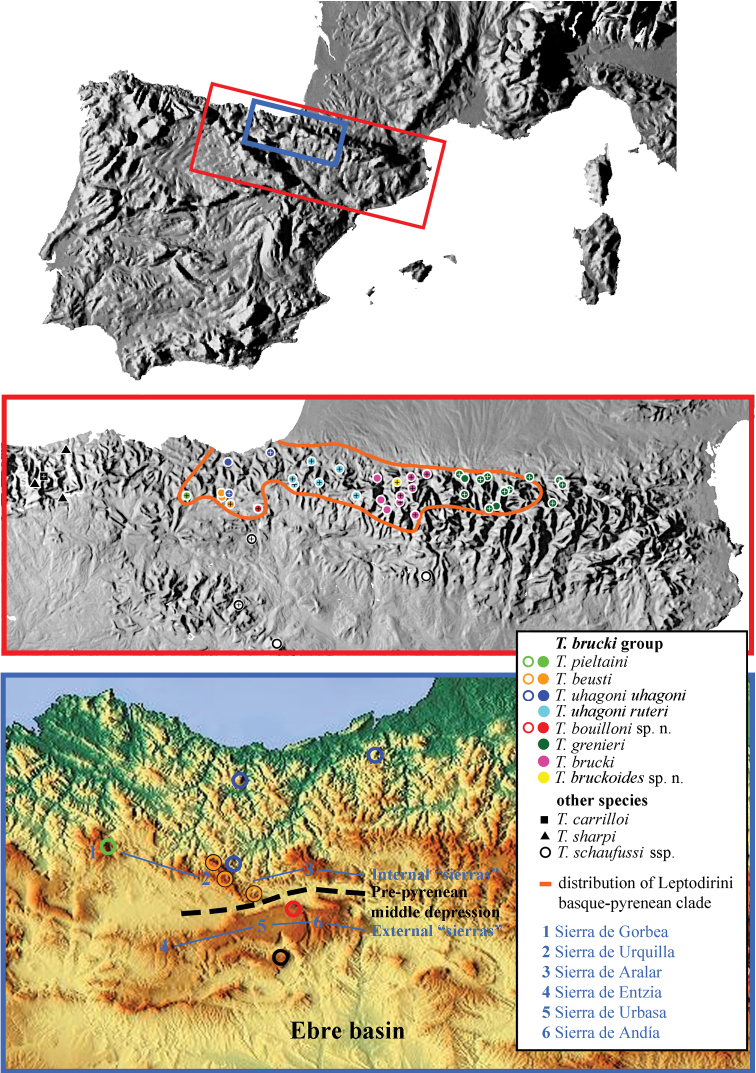
Distribution map of *Trechus brucki* group and related species. Material studied: symbols with cross.

**Figure 36.**
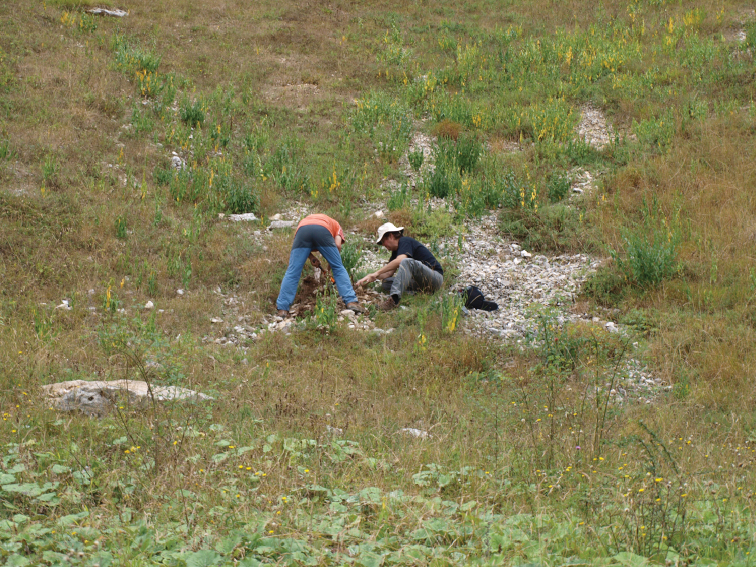
The MSS of Lizarraga pass (Navarra, Spain).

**Figure 37.**
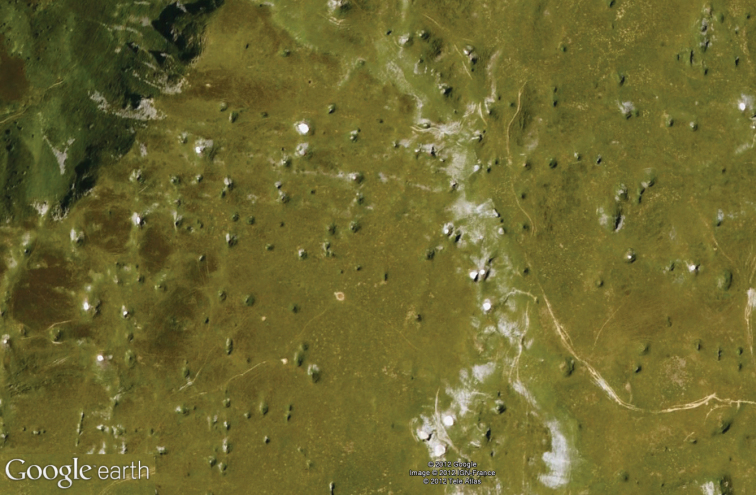
Sinkhole area of the Plateau of Esturou (Hautes–Pyrénées, France).

## Supplementary Material

XML Treatment for Trechus bouilloni

XML Treatment for Trechus brucki

XML Treatment for Trechus bruckoides

## Appendix

Distribution of the *Trechus brucki* group sensu novo: Material studied and bibliographic records (Fig. 35, map). Material not studied indicated by (!)

### *Trechus brucki* group sensu novo

***Trechus beusti* Schaufuss, 1863**

*Anophthalmus beusti* Schaufuss 1863: 149

Spain: Guipúzcoa: **1**. Zegama, Cueva de San Adrián (Schaufuss 1863; Bolívar y Pieltain and Jeannel 1921; Jeannel 1921, 1927; Jeanne and Zaballos 1986; Zaballos and Jeanne 1994; Serrano 2003; Ortuño and Marcos 2003); 4–VIII–2009, Fresneda leg., 1 ♀, voucher number label “ZSM_L199” (ZSM); 14–VIII–2009, Bourdeau and Fresneda leg., 1 ♂, voucher number label “ZSM_L200” (CAF); 31–XII–2009, Bourdeau leg., 4 exx. (CCB); 18–IV–2010, Bourdeau leg., 1 ex. (CCB); 2–V–2011, Bourdeau leg., 1 ♂ and 1 ♀ (CJF), 5 exx. (CCB). **2**. Oñate, cave Iritegui (= Integui) (!) (Serrano 2003; Ortuño and Marcos 2003). **3**. Oñate, cave Tortuga (!) (Serrano 2003; Ortuño and Marcos 2003).

***Trechus bouilloni* Faille, Bourdeau & Fresneda, sp. n.**

See results

***Trechus bruckoides* Faille, Bourdeau & Fresneda, sp. n.**

See results

***Trechus brucki* Fairmaire, 1862**

*Trechus brucki* Fairmaire, 1862a: 548

*Trechus politus* Fairmaire, 1862b (nec *politus* Brullé, 1842): 578

*Trechus planiusculus* Fairmaire, 1862b (nec *planiusculus* Costa, 1858): 578

*Trechus brucki microthorax* Coiffait, 1952: 190

*Trechus brucki pecoudi* Colas & Gaudin, 1935: 248 n. syn.

*Trechus brucki truilheti* Coiffait, 1952: 189

*Trechus brucki vandeli* Coiffait, 1952: 189

France: Pyrénées–Atlantiques: **1**. Val d’Ossau, les Eaux Bonnes (Fairmaire 1862a; Jeannel 1927, 1941). **2.** Sommet du Pic d’Orhy, 1900 m (ssp. *pecoudi*, Colas and Gaudin 1935; Zaballos and Jeanne 1994; in Spain, Navarra, Macizo de Orhí after Serrano 2003); Pic d’Orhy, 5–1925, 1 ♂ and 1 ♀, coll. Nègre (MNHN); Basses Pyrénées, pentes du Pic d’Orry, 29–V–1936, G. Tempère / pierres, 1900–2000 m, 1 ex., coll. Nègre (MNHN), 1 ♂, coll. Coiffait (MNHN). **3.** Pic de Jaout, dans la zone subalpine, vers 1500 m (!) (Jeannel 1941, Bonadona 1971). **4.** Col de Mahourat, au fond de la vallée d´Ossau, près du col du Pourtalet, 1 ♀ (Coiffait 1952); Col du Pourtalet, Basses Pyr, pic de Maourat, alt 2000 m N°776, 28–9–1959, M. LAVIT (Holotype of *Trechus brucki truilheti*, coll. Coiffait, MNHN), 1 ♂ (Paratype of *Trechus brucki truilheti*, coll. Coiffait, MNHN). **5**. Sesques, Lac Isabe, 10–8–1979, 8 exx., Bourdeau leg (CCB). Laruns, Pic de Sesques, bord de névé, 2300 m, 30–VI–2011, 1 ♀, voucher number label “ZSM–L446”, Bourdeau leg. (CAF). **6.** Lescun, 1 ♂ (Coiffait 1952, Bonadona 1971). **7.** Pic d´Anie, 1 ♀ (Coiffait 1952); Pic d´Anie, 1 ♀ bois de Braca, 15–VI–1951, Coll Coiffait (Holotype of *Trechus brucki vandeli*, coll. Coiffait, MNHN). **8.** Pic d’Arlas, 1800 à 2000 m (!) (Jeanne 1984). **9.** Col de la Pierre Saint Martin (!) (Jeanne 1984, in Spain after Zaballos and Jeanne 1994 and Serrano 2003). **10.** Cirque d´Azuns, 1800 m (!) (Jeanne 1984). **11.** Gourette, Lac d’Anglas, 20–8–2000, 6 exx., Bourdeau leg. (CCB). **12.** Jaout, 15–8–1989, 1 ♂, Bourdeau leg. (CCB); 20–VI–1996, 1 ex., Bourdeau leg. (CCB); VIII–1981, 8 exx., Bourdeau leg. (CCB). **13.** Col du Pourtalet, VI–2007, 3 exx., Bourdeau leg. (CCB). Pic d´Anéou, 2000 m, du col du Pourtalet au col d´Anéou, 2000 à 2100 m (Jeanne 1984). Pourtalet, Caperan d´Anéou, 5–VII–2011, 1 ♂, voucher number label “ZSM–L449”, 1 ♀, Bourdeau leg. (CAF). 7–1959, 2 exx, J. Aubry leg (coll. Coiffait, MNHN). **14.** Pène Blanque, 2500 m (!) (Jeanne 1984). **15.** Ossau, Pic de Gaziès, VII–2009, 1 ♀, voucher number label “ZSM–L190”, Bourdeau leg. (CAF). Hautes–Pyrénées: **16.** Arrens, Pic du Gabizos, 2000 m, 10–VII–2010, 1 ♂, voucher number label “ZSM–L329”, 1 ♀, voucher number label “ZSM–L329bis”, Bourdeau leg. (CAF, ZSM), 1 ♀ (CAF). **17.** Pic Granquet, ♂♂ et ♀♀ (Coiffait 1952); Pic Granquet, 1600 m, 5–9–1943, 8 exx. (Holotype and Paratypes of *Trechus brucki microthorax*, coll. Coiffait, MNHN); 1 ex, cotype, 1600 m, 5–9–1943, coll. Nègre (MNHN). Soum de Granquet, Lac d´Ourrec, en haute vallée de l´Esponne (!) (Queinnec and Ollivier 2011).

Spain: Huesca: **18.** Candanchú (!) (Zaballos and Jeanne 1994). After Serrano (2003) in Spain, «Lérida» (mistake), Macizo de Aneu (!).

***Trechus grenieri* Pandellé, 1867**

*Trechus grenieri* Pandellé, 1867: 147

*Trechus bepmalei* Jeannel, 1921: 176

*Trechus despaxi* Jeannel, 1922: 341

*Trechus uhagoni prepyrenaeus* Coiffait, 1974: 24

*Trechus uhagoni aulaensis* Aubry, 1981: 251

France: Hautes–Pyrénées: **1.** Gazost, 1200 m, au bord des ruisseaux en forêt (Pandellé 1867; Jeannel 1927, 1941, Bonadona 1971), 1 ♀, coll. Fairmaire 1906 (MNHN). **2.** Val de Lesponne, sous les amas de feuilles mortes en bordure immédiate des ruisseaux (!) (Bonadona 1971). **3.** Barèges, 1 ♂, ex coll. Jeannel ex coll. Castelnau (MNHN). **4.** Fréchet–Aure, résurgence de la Hèche, 14–V–2008, 1 ♂, voucher number label “ZSM–L13”, 1 ♀, Besson, Bourdeau & Faille leg. (CAF). **5.** Nistos, Bas–Nistos, Grotte de l’Eglise (Bonadona 1971, Corbaz & Jauzion 1988); 2–III–1980, 2 exx., Bourdeau leg (CCB); 27–V–1945: 1 ♂, 17–VI–1945: 1 ♂, coll. Fourès (MNHN); 1 ♂, M. Bouillon rec. (CAF); VI–46, 4 exx., Bourgoin (MNHN); 15–XII–45, 3 exx., Bourgoin Colas (MNHN); 28 exx. (MNHN, coll. Coiffait). **6.** Doline de la Bayelle de Gazave, 6 exx. (MNHN). Haute–Garonne: **7.** Saint–Béat, Cap de Tus, 1200 m, près de la fontaine ferrugineuse au–dessus du col de Couret (Jeannel 1922, 1927, 1941, Bonadona 1971); été 1922, R. Despax, 3 exx., coll. R. Jeannel (MNHN). Saint Béat, Août 1922, 1 ex., coll. Despax in coll. Nègre (MNHN). **8.** Boutx, forêt de Mourtis (Jeannel 1927, 1941, Bonadona 1971). Ft de Mourtis, St Béat, 1450 m, VIII–1926, 9 exx., coll. R. Jeannel, 1931 (MNHN). Ft de Mourtis, 5 exx., coll. Nègre (MNHN). **9.** Arbas, 4–8–1980, 9 exx., Bourdeau leg. (CCB). **10.** Val d’Espingo, 1800 m, au–dessus du lac d’Ôo (!) (Jeannel 1921, 1927, 1941). **11.** Haute vallée du Lys, Superbagnères (Jeannel 1941, Bonadona 1971). Station de Superbagnères, 1650–1700 m, 3–X–1929, 1 ♂, Jeannel (MNHN). « Station de Superbagnères/ pierres 1700 m, 3 oct 1929 », 1 ♂, Gadeau de Kerville, coll. Nègre (MNHN). Ariège: **12.** Rimont, Maison forestière, VII–1962 (*Trechus uhagoni prepyrenaeus*, Holotype ♂, coll. Coiffait MNHN). **13.** Forêt d´Andronne, Le Bosc, vers 1000 m, 1 ex., 2 ♂♂ and 4 ♀♀ (Coiffait 1974). IV–1961, 2 ♂♂, *Trechus uhagoni prepyrenaeus*, paratypes; XI–1961, 3 ♀♀, paratypes (MNHN, coll. Coiffait). **14.** Riverenert, vers 1100 m sur le versant Nord du col de la Crouzette, 2 ♀♀ (Coiffait 1974). La Crouzette, Sentenac de Serou, IX–1960, 2 ♀♀, coll. Coiffait (MNHN). **15.** Lapiaz de Lazur, flanc NE du Mont Valier, VII–1978, 2 exx., Bourdeau leg. (CCB). **16.** Port d’Aula, 2200 m (!) (Aubry 1981). Spain: Lérida: Port d’Aula (!) (Zaballos and Jeanne 1994; Serrano 2003).

***Trechus pieltaini* Jeannel, 1920**

*Trechus pieltaini* Jeannel, 1920: 155

Spain: Álava: **1.** massif of Gorbea (Serrano 2003), Cueva de Mairuelegorreta (Jeannel 1920, 1927, Bolívar y Pieltain and Jeannel 1921, Ortuño and Marcos 2003); 22–V–2011, 1 ♀, voucher number label “ZSM–L395”, Bourdeau leg. (CAF). 22–VII–2011, 4 ♂♂ and 4 ♀♀, Bourdeau leg. (CJF, CAF). 9.IV.1977, 2 exx., Garde leg. (coll. Lagar). **2.** Cueva del Manantial (!) (Bolívar y Pieltain and Jeannel 1921). **3.** Cueva de Arcegui (Monte Gorbea 1000 m, 30TWNI1963), T.M. Zuya–Zuia (!) (Ortuño and Marcos 2003). **4.** Cueva de Sogusti–2 (Monte Gorbea 1000 m, 30TWNI1963), T.M. Zigoitia (!) (Ortuño and Marcos 2003). Vizcaya: **5.** Cueva del Polvorino (= Polvorón) de Elorrea, Ceánuri en el macizo de Gorbea, a 1050 m sobre el nivel del mar (Español 1965, Zaballos and Jeanne 1994); 21–IX–1962, 1 ♀, Nolte leg., coll. Daffner (ZSM). **6.** Ceánuri, Sima A–S–109 (!) (Zaballos and Jeanne 1994).

“Hisp”, “Alte Sammlung”, 3 ♂♂, 1 ♀ (ZSM). We found in the ZSM collection a specimen labelled “Asturien, collection Strasser” which is most probably an erroneous locality.

***Trechus uhagoni*** Crotch, 1869

*Trechus uhagoni* Crotch, 1869: 14

Spain: Navarra: **1**. Zegama, Alsasua (Crotch 1869) Cueva de Orobe (Jeannel 1927; Español 1965, Zaballos and Jeanne 1994; Serrano 2003); 1–VI–2004, 7 exx., Bourdeau leg. (CCB); 13–VII–2004, 1 ♂, voucher number label “MNHN–AF102”, Bourdeau leg. (MNHN), 1 ♀ (CAF); 2–V–2009, 3 exx. (CJF, CAF); 1 ♂, voucher number label “ZSM–L161” Bourdeau and Fresneda leg. (CAF).

We found 4 specimens labelled «Andara Escalera» (MNHN). A locality called Endara exists in SE of Oiartzun (Guipúzcoa). We cannot exclude that the species also occurs in this area. It is also quoted in Guipúzcoa, Macizo de Izarraitz, Ekain (Galán 2003) but the specific attribution of these *Trechus* should be verified.

***Trechus uhagoni ruteri*** Colas, 1935

*Trechus uhagoni ruteri* Colas, 1935: 253

France: Pyrénées–Atlantiques: **1.** Larrau, Cañon d´Holçarté, pont d´Amuby, 7–1934, G. Colas (Holotype, aedeagus not with the specimen (MNHN) (Colas and Gaudin 1935, Jeannel 1941, Bonadona 1971). **2.** Larrau, Bois de Saint Joseph (!) (Jeanne 1984). **3.** Forêt d´Iraty (Jeanne 1984); Iraty, 21–IX–1949, 6 exx., H. Coiffait (MNHN, coll. Coiffait). **4.** Col de Bentarté, près du Mont Urculo, 5–1925, 4 ♂♂ and 1 ♀♀, coll. Jeannel (MNHN) (Jeannel 1941, Bonadona 1971, Jeanne 1984).

Spain: Navarra: **5.** Aoiz, Acueducto de Orbaiceta (Español 1977). **6.** Orbaiceta, Bosque de Irati (Jeanne and Zaballos 1986, Zaballos and Jeanne 1994). **3.** Espinal, puerto de Ibañeta (Zaballos and Jeanne 1994). **7**. Col de Roncevaux, juillet 1934, 2 ♀♀, L. & A. Gaudin (MNHN). 5–1925, 2 exx. (Coll Nègre in MNHN). **8**. Entrada C. de Espinal, C. Bolívar, 2 ♂♂ (MNHN). **6**. Peña Escaori, 1600 m, 5–1925, 1 ♂, Gaudin (MNHN).

Species of uncertain phylogenetic affinities

***Trechus carrilloi* Toribio & Rodríguez, 1997**

*Trechus (Trechus) carrilloi* Toribio & Rodríguez, 1997: 283

Spain: Cantabria: **1.** Campoo de Cabuérniga, Bosque de Saja, UTM 30TUN967728 (Toribio and Rodríguez 1997; Serrano 2003); type series (!) (Toribio and Rodríguez 1997): Holotype ♂, 23–VIII–1997, M. Toribio leg. (CMT); paratypes (CMT, MNCN, col. Carabajal, col. García and col. Rodríguez): 4–X–1997, 11 ♂♂ and 9 ♀♀, J. García leg.; 4–X–1997, 3 ♂♂ and 4 ♀♀, F. Rodríguez leg.; 31–X–1997, 2 ♂♂, M. Toribio leg.; 31–X–1997, 2 ♂♂ and 2 ♀♀, F. Rodríguez leg.; 21–XI–1998, 2 ♂♂, F. Rodríguez leg. Same locality, 27–VIII–2001, 1 ♂, Toribio leg. (CJF).

***Trechus sharpi* Jeannel, 1921**

*Trechus sharpi* Jeannel, 1921: 179

Spain: Cantabria: **1.** Santander, Población, sur le mont Hijedo, au sud–est de Reinosa (Jeannel 1921, 1927; Zaballos and Jeanne 1994). **2.** Santander, Collection Strasser, 1 ♂ (ZSM). **3.** Puerto de San Glorio (Zaballos and Jeanne 1994). **4.** Pico Tres Mares (Zaballos and Jeanne 1994). **5.** Montes cantábricos orientales (Serrano 2003). **6.** Sejo (T.M.Valdaliga), Cva. La Mina, 20–XII–2003, 2 ♂♂, C.G. Luque leg. (Col. Salgado). **7.** Quijas, Cueva la Cuevona, 13–IX–1995, 1 ♂, C.G. Luque leg. (Col. Salgado). **8.** “*Trechus cantaber*”, Monts Cantabriques, Sierra de la Sagra, 3 exx., coll. de la Cruz in coll. Coiffait (MNHN).
